# The Effect of Non-Invasive, Non-Pharmacological Interventions on Autonomic Regulation of Cardiovascular Function in Adults with Spinal Cord Injury: A Systematic Review with Meta-Analysis

**DOI:** 10.1089/neur.2024.0110

**Published:** 2025-01-13

**Authors:** Jacob Schoffl, Ashley Craig, Candice McBain, Ilaria Pozzato, James W. Middleton, Mohit Arora

**Affiliations:** ^1^ John Walsh Centre for Rehabilitation Research, Northern Sydney Local Health District, St Leonards, NSW, Australia.; ^2^ The Kolling Institute, School of Health Sciences, Faculty of Medicine and Health, The University of Sydney, Sydney, NSW, Australia.

**Keywords:** autonomic nervous system, electrical stimulation, exercise therapy, heart rate, psychophysiology, spinal cord injuries

## Abstract

Autonomic regulation of cardiovascular function is often disrupted following a spinal cord injury (SCI). A systematic review was undertaken to evaluate the effect of non-invasive, non-pharmacological (NINP) interventions on cardiovascular autonomic biomarkers in adults with SCI. AMED, CENTRAL, CINAHL EMBASE, and MEDLINE were searched from inception to May 17, 2024. Randomized controlled trials (RCTs) of NINP interventions for cardiovascular autonomic biomarkers (heart rate variability [HRV], systolic blood pressure variability [SBPV], or baroreflex gain) in adults (≥18 years of age) with SCI (>3 months) were included. Primary outcomes included HRV (low-frequency power [HRV-LF], high-frequency power [HRV-HF], root mean square of successive differences [RMSSD]), SBPV (low-frequency power [SBPV-LF]), and baroreflex sensitivity. The quality and certainty of the evidence were assessed using version 2 of the Cochrane risk of bias tool and the Preferred Reporting Items for Systematic Reviews and Meta-Analysis tool, respectively. Of 2651 records identified, six RCTs were included (participants, *n* = 123). HRV-LF (four studies; participants, *n* = 69) and HRV-HF (five studies; participants, *n* = 93) showed no to small changes in favor of NINP interventions ([g = 0.25; 95% confidence interval [CI] = −0.23, 0.73; *p* = 0.31; I^2^ = 0%], [g = 0.00; 95% CI = −0.41, 0.42; *p* = 0.98; I^2^ = 0%], respectively). Limited evidence was available for RMSSD, SBPV-LF, and baroreflex gain. This review found that the evidence is inconclusive regarding the effect of NINP interventions on the included HRV, BPV, and BRS parameters in adults with SCI. Further research with strong methodological rigor is needed to provide greater insights in this area.

## Introduction

A spinal cord injury (SCI) disrupts connections between supraspinal centers and spinal cord circuits, resulting in impaired autonomic regulation. This impairment can have a detrimental impact on cardiovascular function and lead to several challenging health conditions, including autonomic dysreflexia (AD) and orthostatic hypotension (OH).^1^ In higher level injuries (i.e., at or above the sixth thoracic segment [T_6_]), these complications tend to be more severe.^2^ For example, injuries at and above T_6_ can affect cardiac sympathetic innervation (T_1_–T_4_) and supraspinal control of the thoracolumbar segments (above T_1_), contributing to poor blood pressure and heart rate regulation.^3,4^ In addition to AD and OH, impaired autonomic regulation is potentially related to cognitive impairment^5^ and fatigue,^6^ and can lead to the development and progression of cardiovascular diseases, such as heart failure.^7^ These secondary health conditions have a detrimental effect on quality of life and can contribute to a significant financial burden.^8,9^ As such, it is no surprise that restoration of autonomic function ranks as a high priority for recovery among populations with SCI.^10^

However, quantifying the extent of this impaired cardiovascular autonomic regulation remains a challenge. While current clinical assessment tools are available to identify a sensorimotor level of injury^11^ and to document remaining autonomic function,^12^ there is no current gold standard method of quantitatively assessing cardiovascular autonomic dysfunction following an SCI.^13^ Such an assessment is needed given the degree of injury to autonomic pathways seems to not always correlate to the degree of injury to sensorimotor pathways.^13,14^

Assessments that are specific, accessible, and impose minimal burden on patients are promising candidates. Heart rate variability (HRV), blood pressure variability (BPV), and baroreflex sensitivity (BRS) can fit these criteria and provide valuable insights into autonomic regulation, particularly cardiovascular function. These measures can differ between able-bodied (AB) and SCI populations as well as between high- and low-level SCI reflecting, amongst other factors, changes in autonomic regulation of cardiovascular function.^15–18^ For example, the absolute power of the low-frequency component of HRV (HRV-LF) and systolic BPV (SBPV-LF) are often reduced in adults with SCI, particularly higher level injuries, compared with that of AB populations.^13,19^ HRV-LF may reflect a combination of both parasympathetic and sympathetic activity and is associated with baroreceptor activity.^20^ SBPV-LF is believed to reflect the sympathetic innervation of smooth muscle vasculature and is regarded as one of potential markers of autonomic completeness in adults with SCI.^13,15^ In contrast, the root mean square of successive differences (RMSSD) and high-frequency power (HRV-HF) reflect parasympathetic activity, with RMSSD typically used to estimate the regulatory effect of the cardiac vagal nerve.^21^ It is unclear whether these parameters differ between AB and SCI populations.^16,22,23,24^ Collectively, these parameters provide insight into the impaired autonomic regulation of cardiovascular function in adults with SCI and provide a unique target for therapeutic interventions.

While an SCI and its subsequent neuroanatomical changes significantly affect cardiovascular autonomic biomarkers, alterations in psychosocial health may also affect these biomarkers, particularly HRV. In AB populations, pain,^25^ depression,^26^ and cognitive function^27^ are related to resting HRV. In adults with SCI, similar relationships have been established, especially with fatigue, emotional regulation, and pain.^16,28,29^ These relationships support the neurovisceral integration model,^30^ where the prefrontal cortex regions responsible for emotional and cognitive regulation communicate bidirectionally with subcortical-autonomic structures.^31^ In this model, HRV is assumed to reflect these connections and is influenced, to a degree, by cognitive and emotional activity.^30,31^ As such, interventions that target cardiovascular autonomic function may have the potential to benefit not only cardiovascular function but also cognitive and psychosocial health in adults with SCI.

Non-invasive, non-pharmacological (NINP) interventions are a group of therapies that have demonstrated modulatory effects on HRV, BPV, and BRS parameters across different populations.^32,33,34^ They include any intervention that does not involve breaking the skin or the use of medications and can include therapies such as exercise, biofeedback, transcutaneous electrical stimulation, cognitive behavioral therapy, and mindfulness therapies. These interventions are associated with minimal side-effects and are valuable alternatives to surgery and pharmacological interventions.^35,36^

NINP therapies may target sympathetic and/or parasympathetic dysregulation. Following a high-level injury (i.e., at and above T6), cardiovascular dysfunction often occurs as result of disrupted sympathetic pathways, leading to neuroplastic changes at a synapse, cellular, and circuit level.^4^ Therapeutic NINP interventions can aim to target these neuroplastic changes and improve sympathetic regulation. For example, transcutaneous spinal cord stimulation (TSCS) has been shown to reduce sympathetic activity and the severity of AD during a digital anorectal stimulation in an adult with a chronic C_4_ motor-complete SCI.^37^ In another example, during an orthostatic challenge, TSCS has been shown to mitigate the reduction in sympathetic activity via the excitation of sympathetic preganglionic neurons and alleviate symptoms of OH.^38^ Alternatively, NINP interventions may also aim to target parasympathetic regulation. While cardiovascular parasympathetic innervation remains intact following an SCI, HRV parasympathetic parameters can differ between AB and SCI populations^39^ and are associated with fatigue, pain, and emotional regulation.^16,28,29^ Interventions, such as visual stimulation, can aim to induce relaxation and improve HRV parasympathetic parameters in adults with SCI.^40^ As cardiovascular autonomic regulation contributes to various complications following an SCI, the effect of NINP interventions needs to be evaluated.

The objective of this systematic review was to evaluate the effect of NINP interventions on cardiovascular autonomic biomarkers in adults with SCI. While the effect of exercise on HRV and BPV in adults with SCI has been evaluated in previous reviews with inconclusive findings,^41,42^ no review has yet evaluated the effect of all NINP interventions on these biomarkers and included only randomized controlled trials (RCTs). Interventions that target cardiovascular autonomic dysfunction have the potential to alter the course of these associated complications, such as AD and OH, and ultimately improve quality of life. This review aims to contribute the highest level of evidence regarding the effect of NINP interventions on cardiovascular autonomic function in adults with SCI.

## Materials and Methods

A systematic review with meta-analysis was conducted following the framework of the Cochrane Handbook for Systematic Reviews of Interventions^43^ and the Preferred Reporting Items for Systematic Reviews and Meta-Analysis (PRISMA) guidelines.^44^ The protocol for this review was prospectively registered (December 2, 2022) on The International Prospective Register of Systematic Reviews (PROSPERO) (CRD42022370484).

## Eligibility Criteria

### Study design

The inclusion criteria for the review are presented in Table 1. Only published RCTs with parallel-group, within-participant, or cross-over designs were included. When conducted in the appropriate context with methodological rigor, RCTs can provide high-quality evidence for assessing therapeutic effectiveness.^45^ Only studies published in English language were included, as a previous review found no differences when studies in languages other than English were included in systematic reviews for conventional medicine (i.e., drug, radiation, or surgery interventions).^46^

**Table 1.** PICOS Criteria for Inclusion

PICOS	Inclusion criteria
Population	Adults (≥18 years of age) with an SCI (≥3 months post-injury)
Intervention	Single or multiple NINP intervention sessions
Comparison	Usual care, waitlist control, or placebo/sham group
Outcomes	HRV (RMSSD, HRV-LF, HRV-HF), BPV (SBPV-LF), and BR gain.
Study type	Randomized trials (parallel group, within-participant, and cross-over designs)

BPV, blood pressure variability; BR, baroreflex; HF, high-frequency power; HRV, heart rate variability; LF, low-frequency power; NINP, non-invasive, non-pharmacological; RMSSD, root mean square of successive differences; SBPV, systolic blood pressure variability; SCI, spinal cord injury.

### Participants

Studies were included if participants were aged 18 years or older and living with a SCI (at least 3 months post-injury). A study consisting of a mixed condition population (e.g., SCI, traumatic brain injury, and multiple sclerosis) would only be included if the SCI participant data could be extracted separately or >75% of participants had an SCI. No restrictions were placed on cause of injury, level of injury, or completeness of injury to avoid restricting the number of eligible studies.

### Interventions and comparisons

Studies with a NINP therapeutic intervention were included. NINP interventions included, but were not limited to, physical exercise, biofeedback, transcutaneous electrical stimulation, or psychological interventions, such as visualization. No restrictions were placed on the duration, frequency, or intensity of the NINP intervention. Control groups included usual care, waitlist control, or placebo/sham. In studies with more than one intervention group, in addition to a control group, intervention groups would be divided and compared separately to the control group. For example, if there were three groups, intervention A, intervention B, and control, the study would be split into intervention A compared to control and intervention B compared to control.

### Outcomes

Studies were eligible if they included at least one primary outcome. Primary outcomes for this review included the following cardiovascular autonomic parameters: (i) RMSSD; (ii) HRV-LF; (iii) HRV-HF; (iv) SBPV-LF; and (v) baroreflex (BR) gain (Table 2). These outcomes were selected to provide an insight into the two divisions of the cardiovascular autonomic nervous system (ANS): (i) parasympathetic (RMSSD, HRV-HF, and BR gain) and (ii) sympathetic (SBPV-LF and BR gain). Depending on the method of measurement, BR gain can provide insights into both cardiovagal (parasympathetic) and sympathetic activity.^17^ HRV-LF was included as a primary outcome as it can differ between adults with SCI and an AB population^15,16^ and is a complementary measure to HRV-HF. Systolic BPV low-frequency power (SBPV-LF) was included as a primary outcome as it is a potential marker of peripheral sympathetic cardiovascular activity in adults with SCI.^13,15^ It is important to note that these parameters are indirect measures of the ANS regulation and can be affected by other factors beyond cardiovascular sympathetic and parasympathetic activity.

**Table 2.** Description of Primary Cardiovascular Autonomic Outcomes

Primary outcomes	Metric	Description	Believed interpretation
Heart rate variability (HRV)	RMSSD	Root mean square of successive differences	RMSSD is used to estimate vagally mediated changes (parasympathetic activity).^47^ This index is highly correlated with HRV-HF.^48^
	HRV-LF	Low-frequency power	Frequency range of 0.04–0.15 Hz. HRV-LF may reflect both sympathetic and parasympathetic activity.^21^ HRV-LF has also been suggested to reflect baroreflex activity and vasomotor tone.^20^
	HRV-HF	High-frequency power	Frequency range of 0.15–0.4 Hz. HRV-HF reflects parasympathetic activity^21^ and is significantly affected by respiration rate.^21^
Systolic blood pressure variability (SBPV)	SBPV-LF	Low-frequency power	Sympathetic innervation of smooth muscle vasculature. In adults with SCI, SBPV-LF has been shown to be a potential method of quantitatively assessing sympathetic cardiovascular autonomic function.^13^
Baroreflex sensitivity (BRS)	BR gain	Baroreflex gain	Measure of the change in RR interval as a function of a change in blood pressure.^49^ BRS reflects the ability of autonomic effectors to respond to acute changes in blood pressure. Greater BRS (baroreflex gain) is associated with improved regulation of blood pressure, as a sensitive baroreflex can elicit faster and greater responses to acute changes in blood pressure.^17^

BR, baroreflex; HF, high-frequency power; HRV, heart rate variability; LF, low-frequency power; RMSSD, root mean square of successive differences; SBPV, systolic blood pressure variability; SCI, spinal cord injury.

Any validated method for assessing HRV and BR gain was included. For SBPV, studies were only included if they assessed SBPV using continuous beat-to-beat measurements. No restrictions were placed on any assessment parameter, such as participant position, duration of assessment, or task performed during the assessment (e.g., orthostatic challenge, Valsalva maneuver). No restrictions were placed on the derivation of the outcome, including method of preprocessing, cleaning, or calculation.

These parameters display significant interindividual variance and as such within-group analyses were preferred. The mean difference between pre- and post-intervention measurements was used for analyses and where this was not available and unable to be calculated, post-intervention measurements were used. In studies with multiple observations for an individual (i.e., baseline, post-intervention, and 6-month follow-up), data collected immediately at the start and end of the intervention period for all analyses were extracted. For studies that reported multiple outcome measures for the same outcome (i.e., HRV-LF using raw units, logarithmic transformation, or normalized units), raw and/or log-transformed units were preferred for analyses. If a study reported both raw and logarithmically transformed units, the more frequently reported measure across the included studies was used in analyses. Normalized units (nu) are based on the principle of autonomic reciprocity, where sympathetic and parasympathetic activation lie on a continuum.^50^ This does not accurately reflect the dynamic interaction of these branches, which can operate independently, coactively, or reciprocally.^51^

#### Secondary outcomes

Secondary outcomes for this review included: (i) psychosocial measures (mood, pain, fatigue, and quality of life [QoL]); (ii) cost-effectiveness; (iii) consumers’ perception of treatment effectiveness; and (iv) adverse events. A separate search was not conducted for other secondary outcomes.

Adverse events referred to any untoward medical occurrences due to participation in a research study. Guidelines, such as CONSORT, recommend that adverse events be reported for studies that include an intervention.^52^ For this review, adverse events were separated into mild to moderate (i.e., headache and nausea) and severe (i.e., AD), as severe adverse events may be life threatening in a population with SCI.

## Literature Search and Screening

In collaboration with an academic liaison librarian, a high-yield search strategy was developed to address the review objective. This included terms relevant to SCI (e.g., spinal cord injury, paraplegia, and tetraplegia), terms relevant to RCTs (e.g., randomized controlled trial, cross-over trial, double blind, and single blind), and terms relevant to cardiovascular autonomic function (e.g., HRV, BPV, and baroreflex sensitivity) (Supplementary Table S1). No search terms were used for interventions to allow all types of interventions to be captured. Five databases were searched from inception: Allied and Complementary Medicine Database (AMED), Cochrane Central Register of Controlled Trials (CENTRAL), EBSCO CINAHL Plus (CINAHL), Ovid Embase (EMBASE), and Ovid MEDLINE. In addition, backward citation searching was performed on all studies that met inclusion criteria and other reviews conducted in the area. Gray literature and Google Scholar were not searched. The initial search was conducted September 28, 2022, and an updated search was conducted May 17, 2024.

Search results from all databases were merged and exported to Endnote Version 20 (Clarivate, Philadelphia, United States), where duplicates were removed. The remaining studies were uploaded to COVIDENCE (Veritas Health Innovation, Melbourne, Australia, https://www.covidence.org/), where further duplicates were removed. Two review authors (JS and CM/MA) independently screened the titles and abstracts in COVIDENCE to exclude studies that did not matched the inclusion and exclusion criteria. Full-text screening was conducted for those articles that matched or potentially matched the inclusion criteria. Review authors did not screen full-text studies in which they were involved. In such instances, studies were screened by another review author who was not involved in the study. Disagreements between the two review authors were resolved by discussion and arbitrated by a third review author (CM/MA).

## Data Extraction

### Methodology

Two review authors (J.S. and C.M./M.A.) independently performed data extraction for all included studies. Differences between the two review authors were resolved by discussion and, when necessary, arbitrated by a third author (C.M./M.A.). If study data were missing or incomplete, the study authors were contacted via e-mail to obtain the relevant data. If authors did not respond after two attempts at contact or were unable to provide the additional data, whatever data available were included. If insufficient data were available for analyses, only descriptive data were presented in the review. If authors of included studies provided both intention‐to‐treat and per protocol data, intention‐to‐treat data were used. Missing data were not imputed.

### Extracted data

Extracted data from the included studies were compiled into a pre-piloted Excel spreadsheet. Extracted data included the following: (a) study characteristics (i.e., authors, publication year, study setting, and study design [within vs. between-subject design]); (b) characteristics of the study sample (i.e., age, sex, population type, and injury characteristics); (c) intervention used (i.e., type, duration, number of sessions, and intensity); (d) assessment and reporting of outcomes (i.e., method of outcome collection, use of an assessment task, and duration of assessment); and (e) miscellaneous items (i.e., funding, conflicts of interest, and protocol registration) (Supplementary Table S2).

### Issues with data extraction

If data were only provided in graphical form and no response was received from the authors, mean scores and SDs were estimated from the graphs. Where studies did not report SDs, Review Manager 5 ([RevMan 5] Version 5.4.1, Cochrane Collaboration, Copenhagen, Denmark) in-built calculator was used to compute missing SDs if p-values, t-test statistics, sample sizes, and/or the standard error were available.^43^ For cross-over studies, data from the first period of cross‐over were preferred, rather than combined data for subsequent periods. However, if not available, combined data were used. If the study data could not be analyzed correctly, outcome data were extracted and presented in the systematic review but not analyzed as meta-analysis.

## Study Appraisal

### Risk of bias

Version 2 of the Cochrane Risk of Bias tool for randomized trials (RoB 2) was used to assess the bias of included studies. The tool includes five domains for RCTs and an additional domain for cross-over trials. The RoB 2 rates a bias score for each study as one of three outcomes: low risk, some concerns, or high risk. Two review authors (J.S. and C.M./M.A.) assessed the risk of bias in each study, with any differences discussed between authors with a third author (C.M./M.A.) used to settle disagreements.

### HRV assessment tool

An HRV assessment tool was developed to assess the quality and reporting of HRV assessment in the included studies (Supplementary Table S3). This tool was designed using recommendations and tools for the assessment of HRV by Quintana et al., (2016),^53^ Laborde et al., (2017),^54^ Dobbs et al., (2019),^55^ and Catai et al. (2020).^56^ This checklist allowed the reviewers to compare characteristics of HRV assessment between studies and to identify key participant and environmental factors that may help with the interpretation of results. Two authors (J.S. and I.P.) independently evaluated studies using the checklist, with disagreements resolved by discussion and arbitrated by an independent third review author (C.M.) when needed. While the tool is specific to HRV, studies that included SBPV-LF or BR gain were evaluated using this tool as similar assessment details may be reported for these outcomes.

### Meta analyses

Meta-analyses were conducted using RevMan 5 provided there was no significant statistical heterogeneity.^43^ Statistical heterogeneity referred to the extent that the results of the included studies were consistent and was assessed using *I*^2^. Where the *I*^2^ value was (i) <75%, a random‐effects model was implemented and (ii) >75%, the data from studies were not pooled.^43,57^ A random-effects model was chosen as it allows unexplained heterogeneity among studies to be accounted for.^43^ Standardized mean differences were calculated for studies included in the meta-analyses using Hedges’ adjusted g. These were interpreted as small (0.25–0.5), medium (0.5–0.9), or large (≥0.9) based on previous literature.^58^ Hedges g was used as results were not reportedly uniformly across studies (i.e., raw units, log-transformed, and normalized units). Data were presented using forest plots by using the inverse-variance method with pooled data. In addition, clinical and methodological heterogeneities were also considered. Clinical heterogeneity referred to the degree to which factors, namely participant and intervention characteristics, differed between studies. Methodological heterogeneity referred to the degree to which procedures differed between studies, such as the use of blinding, randomization, or measurement of an outcome. Studies were analyzed together given they all aimed to restore and/or modulate autonomic function. Where there was apparent statistical, clinical, and/or methodological heterogeneity, subgroup analyses were taken into consideration.

#### Subgroup analyses

It is acknowledged that various factors influence the effect of interventions on individuals with SCI. As a result, subgroup analyses were planned for primary outcomes to explore the influence of the following variables on effect size: (i) type of NINP intervention (i.e., exercise, stimulation, etc.); (ii) duration of intervention (short term versus long term); (iii) level of injury (paraplegia versus tetraplegia); and (iv) completeness of injury (complete versus incomplete). Sensitivity analyses assessed the robustness of the meta-analyses to the inclusion of studies for the primary outcomes. If 10 or more studies were included in a meta-analysis, a funnel plot was drawn to assess for publication bias.

### Summary of evidence

The Grading of Recommendations Assessment, Development and Evaluation (GRADE) approach was used to evaluate the certainty of the body of evidence as to the level of confidence that an estimate of effect is close to the true value for an outcome.^59^ This approach involved the consideration of within‐trial risk of bias, statistical heterogeneity, directness of evidence, precision of effect estimates, and risk of publication bias.^60^ The certainty of evidence was given an outcome as (i) very low, (ii) low, (iii) moderate, or (iv) high.

## Results

### Search results

**FIG. 1.**
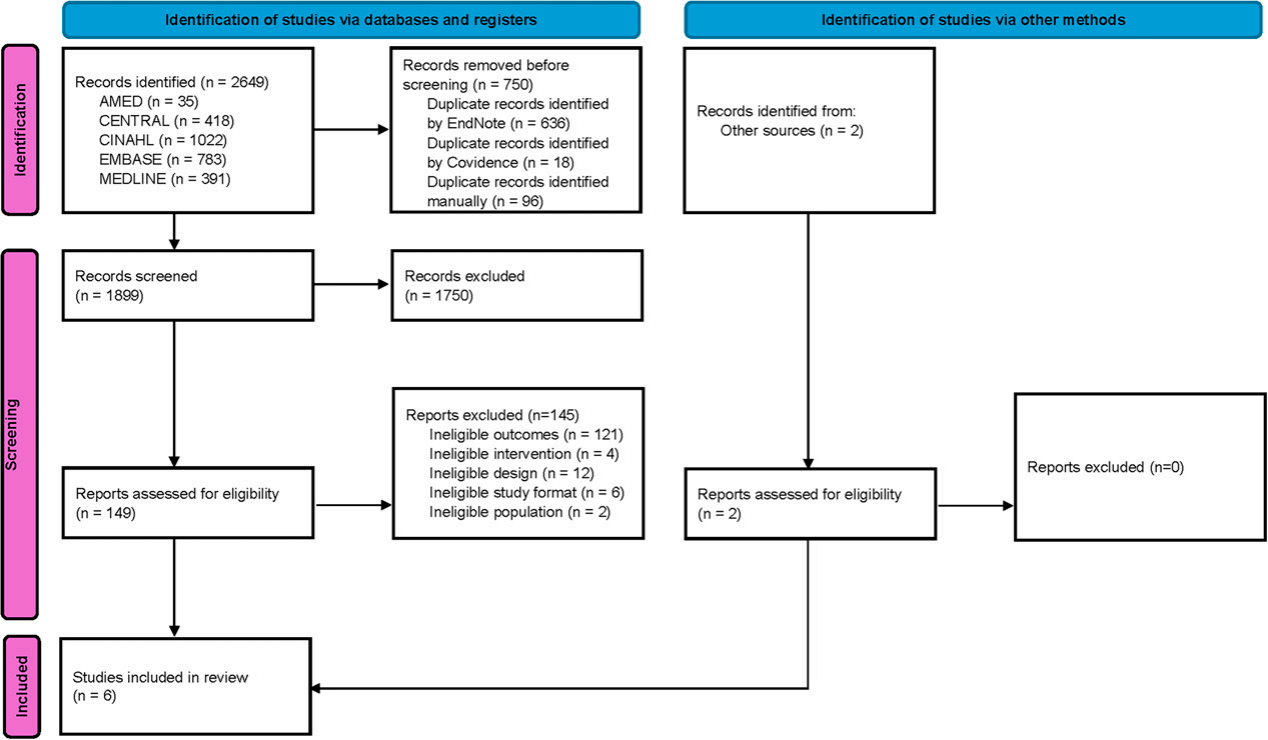
PRISMA flow chart.

In total, 2651 records were identified from databases, registers, and other sources with 1899 undergoing title and abstract screening (Fig. 1). 151 studies underwent full-text screening with six studies included in the final review. Authors of the six included studies were contacted for further information, with replies received from authors of three studies.^18,34,40^ Supplementary Table S4 lists the studies that were excluded during the full-text screening stage and reasons for their exclusion.

### Study characteristics

Table 3 provides a summary of the included studies. Of the six studies, two used a parallel-group design,^18,34^ and the remaining four used a cross-over design.^40,61–63^ All studies were single-centered and conducted in research clinics/laboratories.^18,34,40,61–63^ One study made a prospective protocol available at the time of publication.^34^ Four studies received funding from research grants,^18,34,40,62^ and two studies did not provide information about any source of funding.^61,63^ Two studies declared no conflicts of interest.^34,40^ One study declared the senior author to hold two patents on the method and apparatus used for the intervention.^62^ Three studies did not provide information about any conflicts of interest.^18,61,63^ Studies were undertaken in four different countries Brazil,^61^ France,^63^ Japan,^40^ and the United States.^18,34,62^

**Table 3.** Summary of the Included Studies

	Population	Design	HRV/BPV/BRS protocol (equipment, length, position)	Intervention
Da silva (2017)^61^	SCI (>12 months post) *n* = 18; male = 16; age = 32.9 (7.9); tsi = 16.5 (11.8); complete = 11; T6 = 3; traumatic = 10.	Cross-over	Polar RS800CX, 5-min resting pre- and post-stimulation. Position not reported	Transcranial direct current stimulation vs. sham dose = 1 session; dur = 32 min; protocol = 10 min rest, 12 min stimulation, 10 min rest.
Karri (2018)^62^	SCI (>6 months post) *n* = 10; male = 10; age = 48.2 (13.0); tsi = 13.3 (10.8); complete = 2; T6 = 7; traumatic=NR.	Cross-over	Biocom 5000 wireless ECG, 5-min resting pre–post stimulation, Seated	Breathing combined with electrical stimulation vs. sham dose = 1 session; dur = 120 breaths; protocol=stimulation delivered to forearm on each inhalation.
Ochiai (2017)^40^	SCI (>1 year post) *n* = 24; male = 24; age = 49.0 (16.4); tsi=NR; complete=NR; T6 = NR; traumatic=NR.	Cross-over	ARTETT accelerated plethysmography, 1-min previsualization (resting baseline) and during visual stimulation, Seated	Visual stimulation using a bonsai tree vs. sham dose = 1 session; dur = 1 min; protocol = 1 min of visual stimulation.
Rimaud (2012)^63^	SCI (>2 years post) *n* = 9; male = 9; age = 34.2 (11.5); tsi = 9.9 (9.9); complete = 9; T6 = 4; traumatic = 9.	Cross-over	Holter ECG, 5-min resting pre- and during maximal exercise test, Seated	Use of compression stockings vs. no stockings dose = 1 session; dur=till volitional exhaustion during a maximal exercise test; protocol = 6 min warm up, maximal exercise test, 30 min rest.
Solinsky (2021)^18^	SCI (<24 months post) *n* = 32; male = 29; age = 27.9 (2.6); tsi = 0.87 (0.42); complete = 17; T6 = 27; traumatic = 32.	Parallel	Lead II ECG and finger photoplethysmography (Finapres, Omheda), 3-min resting at baseline and 6 months, Seated	Whole-body exercise training vs. standard of care *n* = 17 participants. dose = 3×/week for 6 months; protocol = rowing involving upper-body pulling and stimulation of lower limb muscles.
Solinsky (2021a)^34^	SCI (>3 months post) *n* = 27; male = 24; age = 28.7 (2.9); tsi = 1.0 (0.1); complete = 16; T6 = 23; traumatic = 27.	Parallel	5-lead ECG and finger photoplethysmography beat-to-beat blood pressure (Finapres, Omheda Medical), 5 cycles of external neck suction, Supine	Whole-body exercise training vs. unstructured exercise *n* = 16 participants. Dose = 2–3×/week for 6 months; dur: 30–60 min each session; protocol = intensive hybrid-FES assisted rowing program.
Solinsky (2021b)^34^	SCI (>3 months post) *n* = 22; male = 20; age = 27.3 (2.4); tsi = 0.8 (0.1); complete = 14; T6 = 18; traumatic = 22.		As above (Solinsky [2021a]).	Arms-only ergometry exercise training vs. unstructured exercise *n* = 11 participants. dose= 2–3×/week for 6 months; dur: 30–60 min each session; protocol = unstructured arms-only ergometry.

AIS, American spinal injury association impairment scale; dur, duration; dur: duration of the intervention; ECG, electrocardiography; FES, functional electrical stimulation; N/A, not applicable; NR: not reported; SCI: spinal cord injury; tsi: time since injury (measured in years); T6: injuries at or above the sixth thoracic segment (measured as the number of participants with injuries at or above T6).

Complete referred to individuals with AIS A or assessed as complete via other means.

### Participants

The total number of participants randomized was 123, with 99 participants analyzed. Ninety-three out of 99 participants were of male sex (93.9%). Data for completeness, level, and etiology of injury were not available for all of the included studies. Forty-four participants had a complete injury (AIS A [44.4%]), 31 participants had an incomplete injury (31.3%), and 24 participants had no reported severity grade (24.3%).^18,34,40,61–63^ Forty-five participants had an injury at or above the level of T_6_ (45.5%), 30 participants had an injury below the level of T_6_ (30.3%), and 24 participants did not have a reported level of injury (24.2%).^18,34,40,61–63^ Fifty-seven participants had an injury due to traumatic causes (57.6%), eight participants had an injury due to non-traumatic causes (8.1%), and 34 participants did not have etiology of injury reported (34.3%).^18,34,40,61–63^ Solinsky 2021a assessed two interventions against a control group and as such was separated into Solinsky 2021a (whole-body exercise compared to control) and Solinsky 2021b (arms-only exercise compared to control), forming a total of seven studies for this review.^34^

### Interventions

The included studies utilized a range of interventions with different hypothesized effects (Supplementary Table S5). The effect of the NINP interventions are presented in Table 4. Da Silva et al. (2017) investigated the acute effect of a single session of transcranial direct current stimulation (tDCS) versus sham stimulation in 18 adults with chronic SCI.^61^ Stimulation (tDCS and sham) was applied for 12 min with an anodal electrode placed over Cz and the reference electrode placed over the occipital tuberosity. Da Silva and colleagues hypothesized that tDCS of the primary motor cortex would restore deafferented sympathetic pathways via top–down modulation.^61^ The study found no significant differences between tDCS and sham sessions for HRV-LF (nu) and HRV-HF (nu).

**Table 4.** Results of the Included Studies

	Intervention	Outcomes	Results used	Findings
da silva (2017)^61^	Transcranial direct current stimulation	HRV-LF (nu) HRV-HF (nu)	Pre–post difference	↔ HRV-LF (nu) and HRV-HF (nu).
Karri (2018)^62^	Breathing combined with electrical stimulation	RMSSD (ms) HRV-LF (ms^2^) HRV-HF (ms^2^) Pain VAS	Post-intervention	↔ HRV-RMSSD (ms), HRV-LF (ms2) and HRV-HF (ms^2^). ↓ Pain VAS.
Ochiai (2017)^40^	Visual stimulation	HRV-HF (ms^2^) POMS	During-intervention	↑ HRV-HF (ms^2^). ↑ POMS-V. ↓ POMS-T, POMS-D, POMS-F, and POMS-TOMD.
Rimaud (2012)^63^	Use of compression stockings	HRV-LF (ms^2^/Hz) HRV-HF (ms^2^/Hz)	Post-intervention	↓ HRV-LF (ms^2^/Hz) and HRV-HF (ms^2^/Hz) post-exercise test for sham and stocking conditions compared to rest. ↔ between groups.
Solinsky (2021)^18^	Whole-body exercise training	HRV-LF (Ln ms^2^) HRV-HF (Ln ms^2^) SBPV-LF (Ln mmHg^2^)	Post-intervention	↔ between groups HRV-LF (Ln ms^2^), HRV-HF (Ln ms^2^), and SBPV-LF (Ln mmHg^2^).
Solinsky (2021a)^34^	Whole-body exercise training	BR gain (ms/mmHg)	Post-intervention	↑ BR gain (ms/mmHg).
Solinsky (2021b)^34^	Arms-only ergometry exercise training	BR gain (ms/mmHg)	Post-intervention	↔ BR gain (ms/mmHg).

BPV, blood pressure variability; BR, baroreflex; ECG, electrocardiography; HF, high-frequency power; HRV, heart rate variability; Hz, Hertz; LF, low-frequency power; mmHg, millimeter of mercury; ms, milliseconds; nu, normalized units; POMS, profile of mood states; POMS-D, depression subscale; POMS-F, fatigue subscale; POMS-TA, tension-anxiety subscale; POMS-TMD, total mood disturbance score; POMS-V, vigor subscale; RMSSD, root mean square of successive differences; SBPV, systolic blood pressure variability; VAS, visual analogue scale.

↔ No change; ↑ Increase; ↓ Decrease.

Karri et al. (2018) investigated the acute effect of a single session of breathing-controlled electrical stimulation (BreEStim) versus sham stimulation in ten male adults with SCI and chronic neuropathic pain (>3 months).^62^ Stimulation (BreEStim and sham) was applied for a total of 120 breaths, with a pair of electrodes placed over the ventral distal forearm. Karri and colleagues hypothesized that BreEStim of the median nerve would modulate brain regions shared between the pain neuromatrix and central autonomic network, aiming to restore autonomic dysfunction (particularly time domain HRV measures of parasympathetic activity implicated in neuropathic pain).^62^ The study found no significant differences between sham and BreEstim conditions for RMSSD, HRV-LF, and HRV-HF. The study also found no significant differences between pre and post measures for both conditions.

Ochiai et al. (2018) investigated the effect of a single session of 1-min visual stimulation versus sham stimulation in 24 male adults with SCI.^40^ Participants were instructed to look at a visual stimulus (bonsai tree [treatment] or empty space [control]) for 60 s. Ochiai and colleagues hypothesized that visual stimulation of the bonsai tree would relax participants.^40^ The study found a significant increase in HRV-HF during visual stimulation of the bonsai tree compared to the control condition.

Rimaud et al. (2012) investigated the effect of graduated compression stockings (GCS) versus no compression during a single wheelchair ergometer test (until volitional exhaustion) in nine male adults with traumatic motor-complete SCI.^63^ Rimaud and colleagues hypothesized that GCS would shift blood flow centrally toward the heart, addressing issues with peripheral vasoconstriction and induce a change in sympathetic activity.^63^ The study found a decrease in HRV-LF and HRV-HF post-exercise for both conditions, but no difference was noted between with and without GCS.

Solinsky et al. (2021) investigated the effect of 6 months of whole-body exercise versus standard of care in 32 adults with SCI (*n* = 17 whole-body exercise, *n* = 15 standard of care [six performing arms-only exercise and nine performing no formal exercise]).^18^ Participants trained between 30 and 60 min, 3×/week for 6 months in either the whole-body exercise (rowing) or standard of care group. Given whole-body exercise can improve HRV and BPV in AB populations, the study investigated whether similar beneficial findings may occur in a population with SCI.^18^ The study found no significant differences between groups for HRV-LF, HRV-HF, and SBPV-LF.

Solinsky et al. (2021a) and (2021b) investigated the effect of 6 months of whole-body exercise and arms-only ergometer training on BR gain in 38 adults with SCI.^34^ Participants were randomized to either a waitlist control, arm-only exercise, and whole-body exercise group. Those in the exercise groups completed exercise 3×/week for 6 months, whereas the waitlist control group completed no formal exercise program. Given whole-body exercise has previously improved BRS in AB populations, the study investigated whether similar findings may occur in a population with SCI.^34^ The study found a significant increase in BR gain post-whole-body exercise, whereas no significant changes were noted for the waitlist controls or arms-only exercise groups.

### Outcomes

Table 4 reports a summary of collected primary and secondary outcomes (excluding adverse events). Meta-analyses were only performed for the outcomes of HRV-LF and HRV-HF due to a limited number of studies. One study reported pre–post (change) scores,^61^ whereas all other studies reported post-intervention scores. This study was included in the meta-analyses and assessed in the high risk of bias sensitivity analysis. The inclusion of this study had no change in the interpretation of results. Publication bias was not assessed for any of the primary outcomes as less than 10 studies were included.

### Primary outcomes

#### HRV-LF

**FIG. 2.**
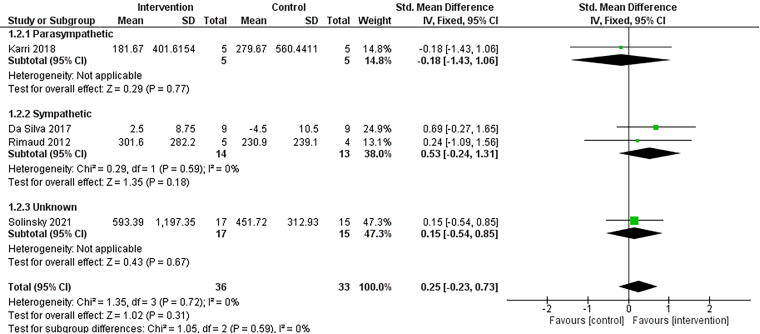
Comparison of non-invasive, non-pharmaceutical interventions vs. control for HRV-LF. Interventions are grouped based on their primary intended effect on the autonomic nervous system. Note: The subgroup analyses presented in this forest plot are based on the hypothesized effect of the intervention on cardiovascular autonomic activity. CI, confidence interval; df, degrees of freedom; HRV-LF, low-frequency component of heart rate variability; IV, inverse variance; SD, standard deviation; Std, standardized.

Four studies reported HRV-LF.^18,61–63^ All studies found no significant differences between groups following NINP interventions.^18,61–63^ Three of the four studies included single-session interventions.^61–63^ Studies that reported HRV-LF (*n* = 4) found NINP interventions to have a small effect in favor of the interventions (Fig. 2, g = 0.25, 95% confidence interval [CI] = −0.23, 0.73, *p* = 0.31, *I*^2^ = 0%). A sensitivity analysis was undertaken for HRV-LF, removing studies with a high risk of bias rating, with no change in the interpretation of the results (*n* = 3, g = 0.10, 95% CI = −0.45, 0.65, *p* = 0.72, *I*^2^ = 0%).

#### HRV-HF

Five studies reported data for HRV-HF.^18,40,61–63^ Four of the five studies found no significant differences between groups following NINP interventions.^18,61–63^ Ochiai et al. (2017) found visualization significantly increased HRV-HF compared to a control condition.^40^ Four of the five studies included single-session interventions.^40,61–63^ Studies that reported HRV-HF (*n* = 5) found NINP interventions to have no effect (g = 0.00, 95% CI = −0.41, 0.42, *p* = 0.98, *I*^2^ = 0%). A sensitivity analysis was undertaken for HRV-HF, removing studies with a high risk of bias rating, with no change in the interpretation of the results (*n* = 3, g = 0.02, 95% CI = −0.53, 0.57, *p* = 0.93, *I*^2^ = 0%).

#### RMSSD, SBPV-LF, and baroreflex sensitivity

Karri et al. (2018) reported data for RMSSD and found no statistically significant differences between (*p* = 0.655, F = 0.20) and within groups for electrical stimulation (*p* = 0.153) versus sham stimulation (*p* = 0.897).^62^ Solinsky et al. (2021) reported data for SBPV-LF and found a non-significant group–time interaction for whole-body exercise versus usual care (*p* = 0.15).^18^ Solinsky (2021a) and (2021b) reported data for BR gain.^34^ Solinsky et al. (2021a) found a significant increase in BR gain for a whole-body exercise group (*p* < 0.05), whereas no change for a waitlist control group (*p* > 0.05). Solinsky et al., (2021b) found no change in BR gain^34^ for both groups (arms-only ergometry and waitlist control, *p* > 0.05).^34^

#### Subgroup analyses

**FIG. 3.**
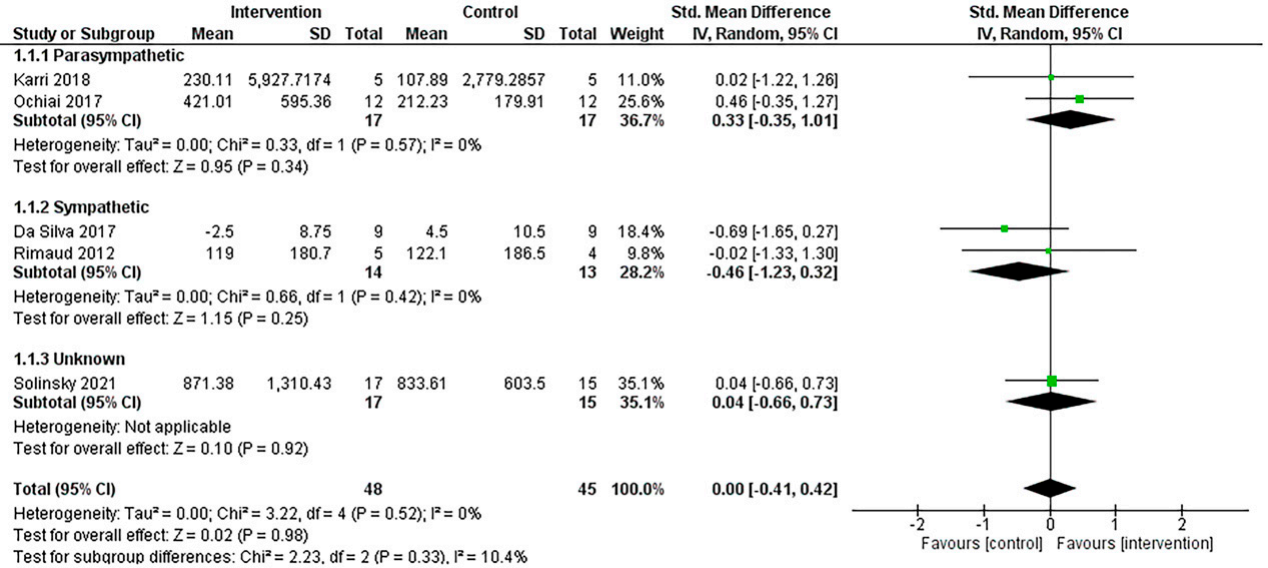
Comparison of non-invasive, non-pharmaceutical interventions vs. control for HRV-HF. Interventions are grouped based on their primary intended effect on the autonomic nervous system. Note: the subgroup analyses presented in this forest plot is based on the hypothesized effect of the intervention on cardiovascular autonomic activty. CI, confidence interval; df, degrees of freedom; HRV-HF, high-frequency component of heart rate variability; IV, inverse variance; SD, standard deviation; Std, standardized.

Subgroup analyses for NINP intervention types, duration of intervention, level of injury, and completeness of injury were not performed as planned due to an insufficient number of studies. However, given the significant clinical and methodological heterogeneity, subgroup analyses were performed based on the hypothesized primary effect of the intervention on either sympathetic or parasympathetic activity. While interventions may target both arms of the ANS, studies were grouped based on their primary intended effect (Figs. 2 and 3). Solinsky et al. (2021) did not present a hypothesized direction of effect for sympathetic and parasympathetic regulation and was placed in an “unknown” effect category.^18^

Two studies were deemed to modulate sympathetic activity.^61,63^ These NINP interventions (*n* = 2) were found to have a moderate effect on HRV-LF in favor of the interventions (g = 0.53, 95% CI = −0.24, 1.31, *p* = 0.18, *I*^2^ = 0%). The addition of Solinsky et al. (2021) reduced the magnitude of effect (g = 0.32, 95% CI = −0.20, 0.84, *p* = 0.22, *I*^2^ = 0% [Supplementary Table S6]). For HRV-HF, these NINP interventions (*n* = 2) were found to have a small effect on HRV-HF in favor of the control condition (g = −0.46, 95% CI = −1.23, 0.32, *p* = 0.25, *I*^2^ = 0%). The addition of Solinsky et al. (2021) reduced the magnitude of effect (g = −0.18, 95% CI = −0.70, 0.33, *p* = 0.49, *I*^2^ = 0% [Supplementary Table S6]).

Two studies were deemed to modulate parasympathetic activity.^40,62^ Only one of these studies evaluated HRV-LF.^56^ For HRV-HF, these NINP interventions (*n* = 2) were found to have a small effect in favor of the interventions (g = 0.33, 95% CI = −0.35, 1.01, *p* = 0.34, *I*^2^ = 0%). The addition of Solinsky et al. (2021) reduced the magnitude of effect (g = 0.18, 95% CI = −0.30, 0.67, *p* = 0.46, *I*^2^ = 0% [Supplementary Table S6]).

### Secondary outcomes

One study reported data on mood changes and fatigue.^40^ The study found a significant decrease in “tension-anxiety,” “depression,” “fatigue,” and total mood disturbance scores, and a significant increase in “vigor” on the Profile of Mood States questionnaire during visual stimulation compared to the control condition.^40^ One study reported data on changes in pain.^56^ The study found a significant reduction in pain levels, measured using a visual analog scale, following direct transcranial current stimulation compared to no change following sham stimulation.^62^ Three studies reported no adverse events occurring during their respective trial.^34,61,62^ Three studies did not report on adverse events.^18,40,63^ None of the included studies reported data for quality of life, cost-effectiveness, or consumer perception of change outcomes.

### Quality assessment

**FIG. 4.**
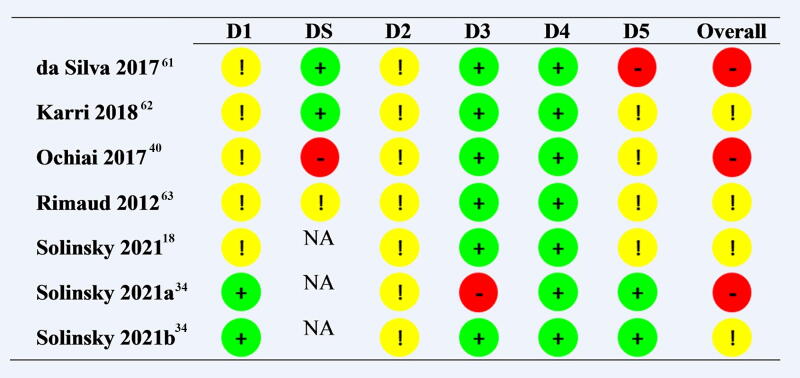
Risk of bias summary for primary outcomes. Review authors’ judgment about each risk of bias item for each included study. D1, randomization process; DS, bias rising from period and carryover effects; D2, deviations from the intended interventions; D3, missing outcome data; D4, measurement of the outcome; D5, selection of the reported result; NA, not applicable. [
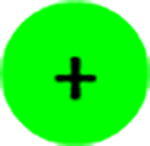
]Low risk; [
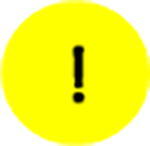
]Some concern; [
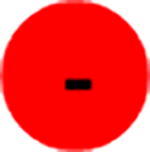
]High risk.

Figure 4 reports a summary of the RoB 2 tool for the seven studies. Of these, four had an overall risk of bias rating of “some concerns,” and three studies had a rating of “high concern.” One study was deemed to have a high risk of bias in the “Selection of the reported result” domain given selective reporting of one outcome using logarithmic transformation.^61^ One study had a high risk of bias in the “Bias rising from period and carryover effects” domain given a lack of wash-out period.^40^ One study had a high risk of bias in the “Missing outcome data” domain given a high proportion of data was missing for the BRS outcome.^34^ Supplementary Table S7 reports the characteristics and detailed RoB 2 assessment for each study.

The HRV assessment tool was used for the five studies that included at least one HRV index^18,40,61–63^ and the one study that reported BR gain (Table 5).^34^ Several items were poorly addressed, including the reporting of respiration, sample size calculation, and the handling of missing data. Some items of the tool were not appropriate for assessing BR gain, and these items were scored as not applicable (NA).

**Table 5.** Heart Rate Variability Assessment Tool

	Da silva (2017)^61^	Karri (2018)^62^	Ochiai (2017)^40^	Rimaud (2012)^63^	Solinsky (2021)^18^	Solinsky (2021a)^34^ & (2021b)^34^	
Population characteristics							
1							
(i) Age	Reported	Reported	Reported	Reported	Reported	Reported	
(ii) Sex	Reported	Reported	Reported	Reported	Reported	Reported	
(iii) Height	Not reported	Not reported	Reported		Reported	Not reported	Not reported
(iv) Weight	Reported	Not reported	Reported		Reported	Not reported	Not reported
(v) Physical activity level	Not reported	Not reported	Not reported	Unclear	Reported	Not reported	
(vi) Alcohol intake	Not reported	Not reported	Not reported	Not reported	Not reported	Not reported	
(vii) Nicotine intake	Not reported	Not reported	Not reported	Not reported	Reported	Not reported	
(vi) Medication status	Reported	Unclear	Not reported	Unclear	Unclear	Not reported	
2							
(i) Neurological level of injury	Reported	Reported	Unclear	Reported	Reported	Reported	
(ii) Time since injury	Reported	Reported	Unclear	Reported	Reported	Reported	
(iii) Sensorimotor completeness of injury	Reported	Reported	Not reported	Reported	Reported	Reported	
(iv) Cause of injury (traumatic/non-traumatic)	Reported	Not reported	Not reported	Reported	Not reported	Reported	
Pre-assessment							
3							
(i) Caffeine	Reported	Not reported	Not reported	Reported	Reported	Reported	
(ii) Alcohol	Reported	Not reported	Not reported	Reported	Reported	Reported	
(iii) Tobacco/illicit substances	Reported	Not reported	Not reported	Reported	Reported	Not reported	
(iv) Bladder/Bowel emptying	Not reported	Not reported	Not reported	Not reported	Not reported	Not reported	
(v) Exercise	Reported	Not reported	Not reported	Reported	Reported	Reported	
(vi) Food	Reported	Not reported	Not reported	Reported	Reported	Reported	
Environment							
4							
(i) Controlled/recorded room temperature	Reported	Not reported	Reported	Not reported	Not reported	Not reported	
(ii) Time of day	Reported	Not reported	Not reported	Reported	Reported	Unclear	
(iii) Position	Not reported	Reported	Not reported	Unclear	Reported	Reported	
(iv) Lighting	Not reported	Not reported	Not reported	Not reported	Reported	Not reported	
Devices							
5							
(i) Name of device/software	Reported	Reported	Reported	Reported	Reported	Reported	
(ii) Configuration of setup	Reported	Reported	Reported	Not reported	Reported	Not reported	
(iii) Sampling rate	Reported	Not reported	Reported	Not reported	Reported	Reported	
Assessment condition							
6							
(i) Stabilization period	Unclear	Reported	Not reported	Reported	Reported	Not reported	
(ii) Length of recording period	Reported	Reported	Reported	Reported	Reported	Reported	
(iii) Respiration (paced/spontaneous)	Not reported	Unclear	Not reported	Not reported	Reported	NA	
Design							
7							
(i) Baseline condition	Reported	Reported	Reported	Reported	Reported	Reported	
(ii) Task/stressor condition	Reported	Reported	Reported	Reported	Not reported	Reported	
(iii) Recovery condition	Reported	Reported	Not reported	Reported	Not reported	Not reported	
(iv) Sample size calculation	Not reported	Not reported	Not reported	Not reported	Not reported	Reported	
(v) Within subject design	Reported	Reported	Reported	Reported	Reported	Reported	
Pre-processing							
8							
(i) Automatic identification of interbeat artifacts	Unclear	Not reported	Reported	Unclear	Reported	Not reported	
(ii) Manual identification of interbeat artifacts	Not reported	Not reported	Not reported	Reported	Reported	Not reported	
(iii) Method of artifact correction	Not reported	Not reported	Not reported	Reported	Not reported	Not reported	
(iv) Percentage of beats corrected	Not reported	Not reported	Not reported	Not reported	Not reported	NA	
(v) Method of spectral analysis (FFT, AR)	Not reported	Reported	Not reported	Reported	Reported	NA	
(vi) Data normality assessment, result, and management	Reported	Reported	Not reported	Reported	Unclear	Not reported	
Analysis							
9							
(i) Length of segment (or number of beats) used in analysis	Reported	Unclear	Reported	Reported	Reported	Reported	
(ii) Software used for preprocessing and identification of interbeat intervals	Reported	Reported	Not reported	Reported	Reported	Not reported	
(iii) Software used for calculation of HRV parameters	Reported	Reported	Not reported	Reported	Reported	Not reported	
(v) Width of frequency bands used for analysis (i.e., LF band [0.04–0.15 Hz])	Reported	Reported	Reported	Unclear	Reported	NA	
(vi) Reasons for data removal	Not reported	Not reported	Not reported	Reported	Not reported	Reported	
(vii) Number of poor quality recordings removed	Not reported	Not reported	Not reported	Not reported	Not reported	Reported	
(vii) Presented as absolute units (can be ln)	Not reported	Reported	Reported	Reported	Reported	Reported	

^a^ Study assesses baroreflex outcome.

AR, autoregressive; FFT, fast-Fourier transform; HRV, heart rate variability; Hz, Hertz; LF, low-frequency; ln, log-transformed; NA, not applicable.

### Certainty of evidence

The certainty of the evidence, the magnitude of the effects of NINP interventions, and the sum of available data for the main outcomes are presented in Table 6. This includes an overall grading of the evidence related to each of the main outcomes using the Preferred Reporting Items for Systematic Reviews and Meta-Analysis approach.^44^

**Table 6.** Certainty of Evidence for Non-Invasive, Non-Pharmacological Interventions Compared to a Control in Adults with Spinal Cord Injury

Population: Adults with an SCI (at least 3 months post-injury) Intervention: Non-invasive, non-pharmacological (NINP) interventions Comparison: Sham/control Outcomes: Cardiovascular autonomic biomarkers (heart rate variability, blood pressure variability, baroreflex gain)								
	Effect		Number of participants		Number of studies	Certainty	Evidence	Importance
Outcomes	Relative (95% CI)	Absolute (95% CI)	Intervention	Control				
HRV-LF —		SMD 0.25 (−0.23 to 0.73)	36	33	4 RCTs	⨁◯◯◯ Very low^a^	It is uncertain if NINP interventions affect HRV-LF compared with sham/control treatment.	Important
HRV-HF	—	SMD 0.00 (−0.41 to 0.42)	48	45	5 RCTs	⨁◯◯◯ Very low^a^	It is uncertain if NINP interventions affect HRV-HF compared with sham/control treatment.	Important
Quality of life	No studies measured quality of life							Important
Participants’ perception of change	No studies measured participants’ perception of change							Critical
Pain	One study measured pain		5	5	1 RCT	NA	—	Critical
Adverse events (mild to moderate)	No adverse events were reported		—	—	3 RCTs	NA	The data were not sufficiently detailed or comparable to be analyzed quantitatively.	Important
Serious adverse events (autonomic dysreflexia)	No studies reported any events of autonomic dysreflexia							Critical

^a^ Downgraded 5 levels: Once for risk of bias—studies that contribute higher weight to the meta-analysis have a high risk of bias criteria. Twice for indirectness—populations, interventions, and outcome assessment methods are all very heterogenous between included studies. Once for imprecision—collective sample size for included studies is low. Once for publication bias—the included studies are at risk of publication bias.

CI, confidence interval; HRV-LF, heart rate variability low-frequency power; HRV-HF, heart rate variability high-frequency power; NINP, non-invasive, non-pharmacological; RCT, randomized controlled trial; SCI, spinal cord injury.

## Discussion

The aim of this review was to evaluate the effect of NINP interventions on cardiovascular autonomic biomarkers in adults with SCI. This review is unique and to our knowledge has not been conducted previously. The review found that the effect of NINP interventions on cardiovascular autonomic biomarkers in adults with SCI is inconclusive. Given the small sample sizes, risk of bias, and other limitations of the included studies, there is very low certainty in the available evidence, and these findings need to be interpreted with caution. Importantly, this review highlights the weak state of current evidence in this field and underscores the need for rigorous studies in the area. To evaluate the effect of NINP interventions on cardiovascular autonomic biomarkers in adults with SCI, greater methodological rigor, larger sample sizes, multiple session interventions, and longer follow-up periods are essential.

In AB populations, HRV is associated with cardiovascular and psychosocial health.^30,31,64^ While associations between psychosocial health and HRV are still present following an SCI, disrupted sympathetic pathways following an SCI provide a layer of complexity to interpreting HRV. There appears to be two rationales for wanting to modulate HRV metrics in this population: (i) to target cardiovascular dysfunction, as a result of dysfunctional sympathetic regulation following an SCI (e.g., injuries at or above T_6_) and (ii) to address neuropsychosocial complications associated with autonomic dysfunction. These two paradigms are reflected by the included studies. For example, Da Silva et al. (2017) and Rimaud et al. (2012) targeted sympathetic regulation, given disrupted sympathetic regulation contributes to a range of autonomic complications.^61,63^ In contrast, Karri et al. (2018) and Ochiai et al. (2021) targetted parasympathetic regulation, given neuropathic pain and mood have been previously linked to reduced parasympathetic HRV parameters.^40,62^ In the subgroup analyses, a larger effect size was seen when separating studies based on their intended targets, rather than when all studies were grouped together. While this is promising, these results do need to be interpreted with caution given the very low sample size and the quality of the studies. These findings highlight the importance of considering the aim of the intervention and intended effect on autonomic modulation when interpreting HRV results and the influence that disrupted sympathetic pathways and psychosocial factors may have on these physiological outcomes.

The lack of conclusive findings in the review may be explained by several reasons. First, the small sample sizes in too few studies greatly undermine the strength of the evidence. As described by Quintana (2017),^58^ to achieve 80% statistical power, sample sizes for HRV studies are recommended to be at least 21, 61, or 233 participants per group in order to detect small, medium, and large effect sizes, respectively. Based on these recommendations, none of the included studies met the requirement to detect even a small effect size. Further, most did not perform power calculations. As such, the included studies were most likely underpowered to be able to clarify the true effect of NINP interventions in an SCI population. Underpowered studies can produce misleading or inconclusive results, waste resources, and lead to problematic interpretations of findings.^65^ Admittedly, recruiting large samples of adults with SCI for neurotherapeutic trials can be challenging.^66^ However, in order to progress this field, larger sample sizes are certainly necessary. The sample sizes provided by Quintana (2017)^58^ are a good starting point, but future studies should also perform transparent sample size calculations based on interventions and outcomes specifically for populations with SCI.

The heterogeneity of SCI populations may also confound the effect of NINP interventions. Factors such as level of injury, time since injury, and extent (sensorimotor/autonomic completeness) of an injury may all affect how an individual responds to an NINP intervention. For example, disrupted bulbospinal input to sympathetic preganglionic neurons occurs in higher level injuries (at and above T_6_) and has been shown to lead to difficulty in controlling heart and vasculature function.^67^ As such, this population may respond differently to an intervention, such as TSCS, than a lower-level injury where cardiovascular impairment may not be as a significant issue. Only two of the included studies discussed the effect of injury level. Solinsky et al. (2021a) found whole-body exercise resulted in significantly greater increases in BR gain for individuals with paraplegia compared to tetraplegia.^34^ In contrast, Da Silva et al. (2017) found that the effect of tDCS was independent of severity, level, and time since injury.^61^ These subanalyses utilized a small sample and must be interpreted with caution. Nonetheless, failing to address such injury heterogeneity may confound the true effect of NINP interventions in this population. Stratification of SCI samples based on injury characteristics and/or the use of specific eligibility criteria is recommended when investigating cardiovascular autonomic function to improve understanding in this area. However, stratifying small samples can further reduce statistical power, highlighting again how insufficient recruitment is a major problem for these studies. Improving recruitment methodologies and developing methodology that accounts for injury heterogeneity will benefit studies in this area.

Similarly, the NINP interventions included in the review were highly heterogeneous. A variety of different types, durations, number of sessions, and intensities were used within the included studies. These variations would all be expected to affect how the intervention modifies autonomic regulation of cardiovascular function. For example, Solinsky (2021a and b) identified duration of training as a mediating factor in their study, reporting that changes in BRS only became apparent after longer periods of exercise training.^34^ Unfortunately, majority of the included studies utilized a single intervention session design, assessing biomarkers either pre–during or pre–post intervention. Using longer-duration studies for these interventions may reveal different findings. This being said, these single sessions utilized a rest–react–recovery design, which are useful to assess how biomarkers respond to these interventions. As suggested by Laborde (2017), using a 3R’s assessment approach (rest–react–recovery) can shed light on phasic HRV, which is often overlooked.^54^ Similarly, Sharif and colleagues suggested a similar approach for adults with SCI, stating that resting values may not be a true reflection of cardiac vagal activity.^68^ They point toward the inclusion of an autonomic stressor during assessments to capture the true integrity of cardiac vagal function, unmasking any dysfunctions that would otherwise remain hidden under resting conditions. Given the above findings, two points are apparent. First, the effect of longer duration interventions needs to be explored, and second, a 3R’s assessment structure is needed to improve assessment of these biomarkers.

Further research is needed to evaluate what constitutes a desirable change in HRV and BPV parameters in adults with SCI. For example, high BPV is undesirable in AB populations given associated organ damage and cardiovascular disease.^69,70^ In contrast, greater SBPV-LF in adults with SCI is correlated with greater sympathetic control of vascular smooth muscle, greater levels of norepinephrine, and reduced symptoms of fatigue and OH.^13,15^ These discrepancies highlight the importance of including injury related and clinical outcomes in studies that use biomarkers, allowing a relationship between changes in biomarkers, injury characteristics, and clinical signs to be established.^71^ As one biomarker does not capture the complete cardiovascular autonomic profile, utilizing combinations of biomarkers beyond the ones included in this review (i.e., skin sympathetic nerve activity, blood and urine catecholamines)^72^ and linking them with clinical symptoms (i.e., AD, OH, fatigue, and pain) and injury characteristics should be encouraged. By incorporating these strategies, a greater understanding of ideal changes for autonomic biomarkers such as HRV and BPV in adults with SCI may be developed, and studies will be better positioned to evaluate the impact of modifying these biomarkers on clinical presentations.

Similarly, studies need to be aware of the high inter- and intra-individual variability that these biomarkers exhibit and utilize study designs that account for this. Using within-participant trial designs rather than between group is recommended for accounting for this inter-individual variability.^73^ Similarly, increasing the frequency of measurements (i.e., continuous or daily measures using wearable devices) may allow individual trends to be captured, rather than collecting a typical pre–post intervention measurement, which is subject to unique daily fluctuations. In a clinical environment, for example, vital biomarkers (i.e., blood pressure, and heart rate) and clinical symptoms are measured regularly throughout a day, allowing for trends to be established and changes in a patient’s health status to be identified. A similar repeated measures design may also be beneficial to apply in studies using HRV, BPV, and BRS, allowing for trends and associations to clinical signs to be established.

There were a few key studies in the area that were excluded from this review because they did not satisfy the inclusion criteria of our review (ineligible study design due to no control arm). Nonetheless, these studies are worth mentioning. Evans and colleagues conducted a parallel RCT (*n* = 16) to evaluate the effect of rehabilitation (3 sessions/week for 24 weeks) using either activity-based therapy or robotic locomotor training on HRV.^74^ They found minimal differences between groups or over time at 6-, 12-, and 24-week assessments under resting and exercise conditions for HRV parameters (standard deviation of R-R intervals and RMSSD). They identified the high variability in HRV as a possible reason for finding no statistically significant results. Similarly, Millar and colleagues conducted a cross-over trial (*n* = 7) to evaluate the effect of body weight supported treadmill training (BWSTT) and head up tilt training (HUTT) (3 sessions/week for 4 weeks) on HRV.^75^ They found no statistically significant effect on frequency domain parameters of HRV when measured under resting conditions, but found that sample entropy, a non-linear measure of HRV, improved after BWSTT but not HUTT. Non-linear parameters aim to capture the quality, scaling, and correlation of the heart signal, rather than the magnitude of variability and may be more sensitive to changes in cardiovascular autonomic function.^75–77^ Despite the small samples included in both studies, their findings are congruent with the overall findings of the current review.

When sample sizes are small, as is the case with most of the included studies, randomization becomes futile.^78^ The aim of randomization is to ensure groups are balanced in terms of overt and hidden baseline characteristics, yet in these small samples, it is unreasonable to expect that baseline characteristics are balanced between groups. As such, cohort and case–control designs are frequently used in this population. These designs complement RCTs, providing larger sample sizes, reduced costs, and can often employ longer follow-up times.^79^ While the internal validity of these designs may not be as rigorous as an RCT, they often have greater scope and generalizability of findings. Several studies using these designs have shown NINP interventions, such as handcycle training,^23^ respiratory training,^80^ and body weight-supported treadmill training,^81^ to have significant effects on cardiovascular autonomic function. However, it should be noted that cohort and case–control studies were deliberately excluded in the current review given the generally held notion that RCTs are the “gold-standard” for evaluating the effectiveness of interventions^82^ and that uncontrolled studies may inflate mean effect estimates more so than controlled trials.^83^ The absence of a control group in such studies introduces several methodological limitations; namely difficultly in establishing the cause-and-effect of an intervention, controlling for confounding factors, and minimizing participant and researcher biases. Nevertheless, a recent Cochrane review found that cohort and case–control studies produce small to negligible differences in effect estimates compared to RCTs.^84^ Given the above, a future review on the topic could consider including cohort and case–control studies to provide a greater understanding as to how NINP interventions affect cardiovascular autonomic biomarkers.

A novel aspect of this review is the use of an HRV assessment tool. While some of the items on the checklist are trivial (i.e., environmental lighting), others are known to have significant effects on results. Several of these items were poorly addressed, including failing to report sample size calculations, respiration rate, and the method of identifying and removing artifacts. Concepts around sample size calculation have been discussed above. Whether to control or not control for respiration during HRV assessment remains unclear. In adults with SCI, HRV-LF and HRV-HF differ between paced and unpaced respiration tasks and can lead to incorrect interpretations if not adequately accounted for.^85^ However, Laborde and colleagues have suggested to account for but refrain from controlling respiration.^54^ They argue that by controlling for respiration, the variance in HRV that occurs naturally from the shared neural origin of HRV and respiration is removed.^54^ While the need to account for respiration or not is outside the scope of this review, studies should clearly report whether respiration was controlled for and the rationale for this decision in order to improve the interpretation of findings.

Additionally, artifacts and missing data can have major implications on HRV calculations with as little as one artifact leading to different HRV results and interpretations.^86^ As such, it is important to communicate the method of artifact cleaning and how missing data were handled. While these issues are of concern, they are not restricted to studies investigating populations with SCI. A review by Grässler and colleagues found that the above items have previously been poorly addressed by studies that investigated the effect of exercise therapy on HRV in AB populations.^32^ Similarly, Kaufmann et al. (2023) reviewed HRV-derived thresholds for exercise prescription in endurance sports for AB populations.^87^ Of the 27 studies reviewed, 37% of studies acknowledged respiration rate and only 4% described how the sample size were derived. It is apparent that these issues are widespread, and a major push in methodological rigor is required. Having transparency in the methodology allows for improved interpretation of results and assists in comparing studies.^53^ Following established guidelines and recommendations, such as those by Quintana et al., (2016)^53^ and Laborde et al. (2017),^54^ will allow studies to be more comparable in the field and assist in understanding the biological mechanisms behind autonomic regulation of cardiovascular function in adults with SCI.

Despite some established links between psychosocial measures and HRV,^6,28^ only a few of the included studies investigated the impact of these interventions on psychosocial health. In the studies that did report psychosocial factors, improvements were found in mood, fatigue, and pain associated with NINP studies.^40,62^ It is well established in AB populations that psychosocial and cognitive health are associated with HRV.^30^ In adults with SCI, the magnitude of these relationships is still being explored, with recent studies finding high correlations between HRV and psychosocial measures, such as anxiety and depression.^28,88^ Future studies investigating these cardiovascular autonomic biomarkers, particularly HRV, should consider including measures of psychosocial health to shed further light on these relationships.

The studies included in the review did not report on cost-effectiveness nor on participants’ perception of change. Cost-effectiveness analysis is valuable for understanding the economic value of an intervention, especially in chronic conditions, such as SCI, which is associated with a high lifetime cost.^9^ Identifying therapies that are cost effective can allow for efficient allocation of healthcare funds to improve access to these treatments, ultimately minimizing the financial burden of these conditions on the individual. Similarly, participants’ perception of change offers insight into how individuals experience an intervention and provides an understanding as to how the course of disease is altered following an intervention. These measures are important for policymakers, funders, and clinicians to assess the “health dollar” value of NINP interventions. Incorporating these outcomes in a mixed method approach to this area of research will provide rich, holistic data to evaluate the clinical utility of these NINP interventions.

There are several strengths of this review. First, this review was unique in identifying and evaluating the effect of NINP interventions on cardiovascular autonomic function in adults with SCI. It employed a rigorous systematic approach for screening studies, including only RCTs, and collated these findings using meta-analyses. The quality of these studies was evaluated using the RoB2 and an HRV assessment tool, identifying key areas where future studies can improve upon.

Nevertheless, there are some limitations with the current review. First, there were a limited number of studies with a small overall sample size. These studies had significant clinical heterogeneity in terms of participant and intervention characteristics, assessment protocols, and comparator groups. This can affect the pooling of results and the findings of this review should be interpreted with this in mind. Second, given the limited number of studies, the effect of individual NINP interventions, level and completeness of injury, and single versus multiple intervention sessions were not evaluated. Pooling these heterogeneous participant and intervention characteristics may hide the true effect of these individual interventions on certain SCI subgroups. Additionally, there were several variations from the registered protocol that occurred during the review, reflecting complexity in the systematic appraisal of studies investigating changes in cardiovascular autonomic function in populations with SCI. These variations and their rationale are presented in Supplementary Table S8. Finally, the HRV assessment checklist is not a validated tool. The tool was designed for evaluating the assessment and reporting of HRV only, not BPV or BR gain. The validity of using the tool for BPV and BR gain has also not been evaluated.

## Conclusion

This review acts as an important step for improving research quality for studies investigating the effect of NINP interventions on cardiovascular autonomic biomarkers in adults with SCI. While it was concluded that the effect of NINP interventions on HRV, BPV, and BRS is inconclusive in adults with SCI, it became clear that further research in this area needs to be adequately powered, assess and report biomarkers appropriately, and follow greater methodological rigor for more definitive conclusions to be made.

## Data Availability

The data that support the findings of this study are available from the corresponding author (JS) upon reasonable request.

## Abbreviations Used

AB: Able-bodied
AD: Autonomic dysreflexia
AIS: American Spinal Injury Association impairment scale
ANS: Autonomic nervous system
BPV: Blood pressure variability
BR: Baroreflex
BRS: Baroreflex sensitivity
CI: Confidence interval
ECG: Electrocardiography
GCS: Graduation compression stockings
GRADE: The Grading of Recommendations Assessment, Development and Evaluation
HF: High-frequency power
HRV: Heart rate variability
Hz: Hertz
LF: Low frequency power
NINP: Non-invasive non-pharmacological
nu: Normalized units
OH: Orthostatic hypotension
RCT: Randomized controlled trial
RMSSD: Root mean square of successive differences
RoB 2: Version 2 of the Cochrane Risk of Bias tool for randomized trials
SBPV-LF: Systolic blood pressure variability low frequency power
SCI: Spinal cord injury
SD: Standard deviation
tDCS: Transcranial direct current stimulation
TSCS: Transcutaneous spinal cord stimulation
