# Supplementary material for: The Effect of Non-Invasive, Non-Pharmacological Interventions on Autonomic Regulation of Cardiovascular Function in Adults with Spinal Cord Injury: A Systematic Review with Meta-Analysis
Source: Neurotrauma Rep. 2025 Jan 13;5(1):1151–72. doi: 10.1089/neur.2024.0110 (PMC11848056; doi:10.1089/neur.2024.0110)
Supplement: Supplementary Table S1 [file neur.2024.0110_supp_table1.docx]

| **Table S1:** Search terms used | |
| --- | --- |
| 1. | exp spinal cord injury/ |
| 2. | spinal cord injur*.mp. |
| 3. | exp paraplegia/ |
| 4. | parapleg*.mp. |
| 5. | exp quadriplegia/ |
| 6. | ((spine or spinal) adj3 (broken or break* or fractur* or wound* or trauma* or injur* or dama* or lesion* or contusion* or laceration* or trauma or ischmi*)).mp. |
| 7. | quadripleg*.mp. |
| 8. | tetraplegi*.mp. |
| 9. | 1 or 2 or 3 or 4 or 5 or 6 or 7 or 8 |
| 10. | exp randomized controlled trial/ |
| 11. | Randomized control trial.mp. |
| 12. | cross-over studies/ or double-blind method/ or single-blind method/ |
| 13. | (random* or factorial* or crossover* or cross over* or cross-over* or placebo* or assign* or allocat* or volunteer*).mp. |
| 14. | (doubl* adj blind*).mp. |
| 15. | (singl* adj blind*).mp. |
| 16. | 10 or 11 or 12 or 13 or 14 or 15 |
| 17. | exp heart rate/ |
| 18. | heart rate.mp. |
| 19. | exp heart rate variability/ |
| 20. | heart rate variability.mp. |
| 21. | HRV.tw. |
| 22. | exp blood pressure/ |
| 23. | blood pressure.mp. |
| 24. | exp blood pressure variability/ |
| 25. | blood pressure variability.mp. |
| 26. | BPV.tw. |
| 27. | baroreflex sensitivity.mp. |
| 28. | BRS.tw. |
| 29. | 17 or 18 or 19 or 20 or 21 or 22 or 23 or 24 or 25 or 26 or 27 or 28 |
| 30. | 9 and 16 and 29 |
| **Note:** *Limits were set on CINAHL to retrieve studies published in English and in adult humans (>18 years of age).* | |
