# Supplementary material for: The Effect of Non-Invasive, Non-Pharmacological Interventions on Autonomic Regulation of Cardiovascular Function in Adults with Spinal Cord Injury: A Systematic Review with Meta-Analysis
Source: Neurotrauma Rep. 2025 Jan 13;5(1):1151–72. doi: 10.1089/neur.2024.0110 (PMC11848056; doi:10.1089/neur.2024.0110)
Supplement: Supplementary Table S2 [file neur.2024.0110_supp_table2.docx]

| **Table S2:** Data extracted for each study | |
| --- | --- |
| Study | author; year of publication; country of study; study setting; study design (within vs. between-subject). |
| Participants: | - inclusion and exclusion criteria; number of males/females; mean age; number of participants recruited/analysed; reasons for withdrawals; time since SCI; neurological level of SCI; completeness of injury; etiology of injury. |
| Intervention: | - mode, duration, intensity, type of intervention and frequency, and type of control (if applicable); device manufacturer (if applicable); details of co-interventions; number of participants randomised in each arm; number of participants analysed in each arm; number of males/females in each arm. |
| Outcomes: | - types of primary outcome(s) and secondary outcome(s); method of assessing outcomes (including any task); time points assessed and used in analysis; summary estimates of primary and secondary outcome(s) for each time point (mean differences with standard deviations [SDs]). |
| Miscellaneous: | - funding source(s) (if applicable); detailed of published protocol (if applicable); disclosures. |
