# Supplementary material for: The Effect of Non-Invasive, Non-Pharmacological Interventions on Autonomic Regulation of Cardiovascular Function in Adults with Spinal Cord Injury: A Systematic Review with Meta-Analysis
Source: Neurotrauma Rep. 2025 Jan 13;5(1):1151–72. doi: 10.1089/neur.2024.0110 (PMC11848056; doi:10.1089/neur.2024.0110)
Supplement: Supplementary Table S3 [file neur.2024.0110_supp_table3.docx]

| **Table S3:** Items of the HRV assessment tool. | | |
| --- | --- | --- |
| **Items** | **Title** | **Explanation of Item** |
| 1 & 2 | Population characteristics | Age, sex, physical activity level, alcohol intake, nicotine intake, and medication status should be reported (Quintana et al., [2016]).  Neurological level of injury, time since injury, sensorimotor completeness of injury, and cause of injury are expected to influence HRV results. As per Quintana et al., (2016), a description of duration of disease and severity should be described. |
| 3 | Pre-assessment | Caffeine, alcohol, tobacco smoking, bladder/bowel emptying, intense physical activity, and food consumption should be reported (Laborde et al., [2017]). |
| 4 | Environment | Temperature, time of day, position of participant, and lighting of assessment area should be reported (Catai et al., [2019]). |
| 5 | Devices | Brand and electrode configuration, acquisition software, and sampling rate should be reported (Quintana et al., [2016]), Catai et al., [2019]). |
| 6 | Assessment condition | Stabilisation period, length of recording period, and acknowledgement of respiration should be reported (Catai et al., [2019]). |
| 7 | Design | The study should include a resting baseline, task, and recovery condition using a within-subject design. Sample size calculations should be reported (Laborde et al., [2017]). |
| 8 | Pre-processing | Method of identification and correction of interbeat artifacts, percentage of beats corrected, method of spectral analysis (FFT, AR), description of HRV parameters (i.e., interpretation), data normality assessment and management should be reported (Quintana et al., [2016], Laborde et al., [2017]). |
| 9 | Analysis | Length of segment, software used for pre-processing and identification of interbeat intervals and calculation of HRV parameters, width of frequency bands used for analysis, reasons for data removal, number of poor-quality recordings removed, and presentation of units should be reported (Laborde et al., [2017], Catai et al., [2019]). |
