# Supplementary material for: The Effect of Non-Invasive, Non-Pharmacological Interventions on Autonomic Regulation of Cardiovascular Function in Adults with Spinal Cord Injury: A Systematic Review with Meta-Analysis
Source: Neurotrauma Rep. 2025 Jan 13;5(1):1151–72. doi: 10.1089/neur.2024.0110 (PMC11848056; doi:10.1089/neur.2024.0110)
Supplement: Supplementary Table S4 [file neur.2024.0110_supp_table4.docx]

| **Table S4:** Characteristics of excluded studies | |
| --- | --- |
| **Reason for exclusion** | **Study ID** |
| Ineligible outcome | Aidar 2022 |
|  | Akkurt 2017 |
|  | Alexeeva 2011 |
|  | Arazpour 2016 |
|  | Ardestani 2019 |
|  | Averill 2000 |
|  | Bailey 2020 |
|  | Bakkum 2015 |
|  | Bakkum 2015 |
|  | Ballaz 2008 |
|  | Bhambhani 1991 |
|  | Borello-France 2000 |
|  | Brazg 2017 |
|  | Brown 2007 |
|  | Burns 2012 |
|  | Butler 2004 |
|  | Champagne 2016 |
|  | Chao 2005 |
|  | Claydon 2006 |
|  | Cooper 2001 |
|  | Cooper 2001 |
|  | Corbianco 2021 |
|  | daCunha-Filho 2003 |
|  | DeGroot 2003 |
|  | DeGroot 2005 |
|  | deGroot 2023 |
|  | Drory 1990 |
|  | Duran 2001 |
|  | Evans 2019 |
|  | Faghri 1992 |
|  | Faghri 2001 |
|  | Faulkner 2021 |
|  | Fornusek 2008 |
|  | Fornusek 2014 |
|  | Forsyth 2016 |
|  | Franek 1988 |
|  | Froehlich-Grobe 2022 |
|  | Gass 2002 |
|  | Gater 2007 |
|  | Goosey-Tolfrey 2008 40 |
|  | Goosey-Tolfrey 2010 |
|  | Graham 2019 |
|  | Hagobian 2004 |
|  | Hasnan 2013 |
|  | Hawkins 2022 |
|  | Hirokawa 1990 |
|  | Hoekstra 2013 |
|  | Holme 2001 |
|  | Hooker 1992 |
|  | Hooker 1995 |
|  | Horiuchi 2017 |
|  | Jacobs 1997 |
|  | Jacobs 2002 |
|  | Jacobs 2002 |
|  | Jacobs 2002 |
|  | Jacobs 2003 |
|  | Jansen 2021 |
|  | Janssen 2008 |
|  | Jung 2012 |
|  | KesiktaÅŸ 2021 |
|  | Keyser 2003 |
|  | Khurana 2016 |
|  | Lamont 1996 |
|  | Larson 2018 |
|  | Laubacher 2015 |
|  | Lavado 2012 |
|  | Lee 2003 |
|  | Levine 2018 |
|  | Lieberman 2014 |
|  | Lin 1993 |
|  | Lopes 1984 |
|  | Lotter 2020 |
|  | Lytle 2019 |
|  | McCulloch 2013 |
|  | McLean 1995 |
|  | McLean 1995 |
|  | Merati 2000 |
|  | Midha 1999 |
|  | Mizushima 2003 |
|  | Mukherjee 2001 |
|  | Mutton 1997 |
|  | Nash 1995 |
|  | Nash 1996 |
|  | Nash 1997 |
|  | Nash 2008 |
|  | Nooijen 2017 |
|  | O'Connor 2002 |
|  | Onushko 2019 |
|  | Ordonez 2013 |
|  | Raymond 1999 |
|  | Raymond 1999 |
|  | Raymond 2002 |
|  | Rimaud 2007 |
|  | Rosety-Rodriguez 2014 |
|  | Rosety-Rodriguez 2014 |
|  | Sabour 2018 |
|  | SahagunOlmosRoberto 2015 |
|  | Sampson 2000 |
|  | Sankari 2015 |
|  | Scheer 2015 |
|  | Schuck 2012 |
|  | Solinsky 2021 |
|  | Solinsky 2023 |
|  | Son 2022 |
|  | Spoljar 2015 |
|  | Stangier 2019 |
|  | Stevens 2015 |
|  | Taylor 1986 |
|  | Taylor 2014 |
|  | TerWoerds 2006 |
|  | Thijssen 2005 |
|  | Thomas 1997 |
|  | Thumwaree 2011 |
|  | Tordi 2001 |
|  | Wadsworth 2011 |
|  | Wouda 2016 |
|  | Yamamoto 1999 |
|  | Yamasaki 2000 |
|  | Yarar 2011 |
|  | Yoshida 2013 |
|  | Yoshida 2013 |
| Ineligible study design | Ballaz 2007 |
|  | Bhambhani 2000 |
|  | Carty 2012 |
|  | Ellaway 2010 |
|  | Evans 2021 |
|  | Garner 1985 |
|  | Hooker 1990 |
|  | Hopman 1998 |
|  | Millar 2009 |
|  | Savage 2021 |
|  | Wecht 2006 |
|  | Yang 2008 |
| Ineligible study format | Coulter 2015 |
|  | Knezevic 2021 |
|  | McCormack 2012 |
|  | Vogiatzi 2015 |
|  | Wecht 2019 |
|  | Wu 2019 |
| Ineligible intervention | Chen 2004 |
|  | Faghri 2002 |
|  | Flueck 2016 |
|  | Wecht 2017 |
| Ineligible patient population | Fann 2003 |
|  | Karri 2021 |
