# Supplementary material for: The Effect of Non-Invasive, Non-Pharmacological Interventions on Autonomic Regulation of Cardiovascular Function in Adults with Spinal Cord Injury: A Systematic Review with Meta-Analysis
Source: Neurotrauma Rep. 2025 Jan 13;5(1):1151–72. doi: 10.1089/neur.2024.0110 (PMC11848056; doi:10.1089/neur.2024.0110)
Supplement: Supplementary Table S5 [file neur.2024.0110_supp_table5.docx]

| **Table S5:** Hypothesised direction of effect. | | | |
| --- | --- | --- | --- |
|  | **Intervention** | **Outcomes** | **Hypothesised direction of effect** |
| **da Silva**  **(2017)** | Transcranial direct current stimulation | HRV-LF (nu)  HRV-HF (nu) | **Authors’ hypothesis:** “Our hypothesis is that motor cortex stimulation will be able to restore defective pathways (deafferentation of the sympathetic sensorial pathways) via top-down modulation, thus allowing the modulation of the autonomic nervous system in SCI patients.”  **Reviewers’ perspective:**  Whilst no direction of change was specified for HRV parameters, a **change in sympathetic parameters** was most likely intended. |
| **Karri (2018)** | Breathing combined with electrical stimulation | RMSSD (ms)  HRV-LF (ms^2^)  HRV-HF (ms^2^) | **Authors’ hypothesis:** “Essentially, we aimed to explore the utility of HRV differences to capture acute autonomic responses to treatment of chronic NP following SCI.”  “We hypothesized that centrally directed NP analgesia via BreEStim would affect PNM–CAN activity, and thus HRV measures, in persons with chronic NP following SCI. As such, BreEStim-induced analgesia was hypothesized to be accompanied by restoration of automatic dysfunctions in these persons with NP following SCI as seen in our study.^24^ In particular, we hypothesized that those HRV parameters produced by time domain analysis would be sensitive to parasympathetic tone changes associated with NP and with BreEStim treatment for NP.”  **Reviewers’ perspective:**  Whilst no direction of change was specified for HRV parameters, a **change in parasympathetic parameters** was most likely intended. The referenced article identified reduced SDNN as a measure of NP in adults with SCI + NP vs. SCI with no NP. |
| **Ochiai (2017)** | Visual stimulation | HRV-HF (ms^2^) | **Authors’ hypothesis:** “One-sided tests were used because we hypothesized that the patients would be relaxed after viewing the bonsai trees.”  **Reviewers’ perspective:**  A **change in parasympathetic parameters** was most likely intended. |
| **Rimaud (2012)** | Use of compression stockings | HRV-LF (ms^2^)  HRV-HF (ms^2^) | **Authors’ hypothesis:** “The purpose of the present study was, therefore, to investigate if routinely prescribed GCS in SCI could affect sympatho-adrenergic activity and HRV at rest and after a strenuous wheelchair exercise in individuals with SCI.”  **Reviewers’ perspective:**  Whilst no direction of change was specified for HRV parameters, a **change in sympathetic** **parameters** was most likely intended. |
| **Solinsky (2021)** | Whole-body exercise training | HRV-LF (Ln ms^2^)  HRV-HF (Ln ms^2^) | **Authors’ hypothesis:** “since exercise training is associated with beneficial changes in HRV and BPV, we determined whether these specific improvements could be realized in individuals with SCI.”  **Reviewers’ perspective:**  Whilst no direction of change was specified for HRV parameters, the authors discuss how low HRV and high BPV can be detrimental in other populations and SCI populations generally have lower HRV. We assume an **increase in HRV parameters** was most likely intended, however this should be interpreted with caution. |
| **Solinsky (2021a)** | Whole-body exercise training | BR gain (ms/mmHg) | **Authors’ hypothesis:** “Given the crucial importance of blood pressure regulation in these individuals, our objective was to determine the effects of 6 months of high-intensity, whole-body exercise compared to standard of care on cardiovagal baroreflex sensitivity in individuals with SCI.”  **Reviewers’ perspective:**  Whilst no direction of change was specified for BR gain, the authors discuss how aerobic exercise training has previously improved BRS. We assume an **increase in BRS** was most likely intended. |
| **Solinsky (2021b)** | Arms-only ergometry exercise training | BR gain (ms/mmHg) | No hypothesis was specified for arms only ergometry. |
| BPV: blood pressure variability; BR: baroreflex; BreEStim: breathing combined with electrical stimulation; BRS: baroreflex sensitivity; CAN: central autonomic network; ECG: electrocardiography; GCS: graduated compression stockings; HF: high frequency power; HRV: heart rate variability; int: intervention; LF: low frequency power; Ln: log transformation; mmHg: millimetre of mercury; ms: milliseconds; NN50: number of pairs of successive NN (R-R) intervals that differ by more than 50 ms; pNN50: proportion of NN50 divided by the total number of NN (R-R) intervals; NP: neuropathic pain; nu: normalised units; PNM: pain neuromatrix; RMSSD: root mean square of successive differences; SBPV: systolic blood pressure variability; SCI: spinal cord injury; tDCS: transcranial direct current stimulation. | | | |
