# Supplementary material for: The Effect of Non-Invasive, Non-Pharmacological Interventions on Autonomic Regulation of Cardiovascular Function in Adults with Spinal Cord Injury: A Systematic Review with Meta-Analysis
Source: Neurotrauma Rep. 2025 Jan 13;5(1):1151–72. doi: 10.1089/neur.2024.0110 (PMC11848056; doi:10.1089/neur.2024.0110)
Supplement: Supplementary Table S6 [file neur.2024.0110_supp_table6.docx]

| **Table S6:** Sensitivity analysis for Solinsky (2021) | | | |
| --- | --- | --- | --- |
| **Outcome** | **Intended effect** | **All studies (with Solinsky [2021])** | **Without Solinsky (2021)** |
| HRV-LF | Sympathetic | g=0.32, 95% CI=-0.20, 0.84, p=0.22, I^2^=0% | g=0.53, 95% CI=-0.24, 1.31, p=0.18, I^2^=0% |
|  | Parasympathetic | g=0.07, 95% CI=-0.53, 0.68, p=0.81, I^2^=0% | NA (n=1) |
| HRV-HF | Sympathetic | g=-0.18, 95% CI=-0.70, 0.33, p=0.49, I^2^=0% | g=-0.46, 95% CI=-1.23, 0.32, p=0.25, I^2^=0% |
|  | Parasympathetic | g=0.18, 95% CI=-0.30, 0.67, p=0.46, I^2^=0% | g=0.33, 95% CI=-0.35, 1.01, p=0.34, I^2^=0% |
| CI: confidence interval; HF: High frequency power; HRV: Heart rate variability; LF: Low frequency power; NA: not applicable. | | | |
