# Supplementary material for: The Effect of Non-Invasive, Non-Pharmacological Interventions on Autonomic Regulation of Cardiovascular Function in Adults with Spinal Cord Injury: A Systematic Review with Meta-Analysis
Source: Neurotrauma Rep. 2025 Jan 13;5(1):1151–72. doi: 10.1089/neur.2024.0110 (PMC11848056; doi:10.1089/neur.2024.0110)
Supplement: Supplementary Table S7 [file neur.2024.0110_supp_table7.docx]

| **Table S7:** Risk of bias assessment for the included studies | | | |  |
| --- | --- | --- | --- | --- |
| ***Risk of bias*** | *Da Silva (2017)* |  |  |  |
| **Domain** | **Signalling** | **Judgement** | **Support for judgement** | **Domain risk of bias** |
| 1. Randomisation process | 1.1 Random allocation sequence | NI | Quote: “Subjects were then randomized to either sham or active tDCS.” See “2.3 Experimental procedures”, pg. 161.  Comment: Insufficient detail regarding random allocation sequence. | Some concerns |
|  | 1.2 Allocation concealment | NI | Comment: No detail regarding allocation concealment. |  |
|  | 1.3 Baseline differences | N | Comment: Baseline differences do not suggest an issue in the randomisation process. |  |
| S. Period and carryover effects | S.1 Was the number of participants allocated to each of the two sequences equal or nearly equal? | Y | Comment: n=10 and n=9 started with the stimulation and sham conditions respectively. | Low |
|  | S.2 Were period effects accounted for in the analysis? | NA | - |  |
|  | S.3 Was there sufficient time for any carryover effects to have disappeared before outcome assessment in the second period? | Y | Comment: 72-hours appears to be sufficient time to avoid carry-over effects between stimulation sessions. Justification for this time length is supported by a citation in the article. See “2.3 Experimental procedures”, pg. 161. |  |
| 2. Deviations from intended interventions | 2.1 Participants aware of assigned intervention | PN | Comment: The authors state that the trial is double-blinded with sham and active treatments but does not state who is blinded. The review team assume participants and assessors are blinded. | Some concerns |
|  | 2.2 Carers/people delivering interventions aware of assignment | PY | Comment: As above. |  |
|  | 2.3 Deviations from intended intervention due to trial context | NI | Comment: No mention of deviations from intended interventions due to trial context. |  |
|  | 2.4 Were these deviations likely to have affected the outcome? | NA | - |  |
|  | 2.5 Were these deviations from intended intervention balanced between groups? | NA | - |  |
|  | 2.6 Was an appropriate analysis used to estimate the effect of assignment to intervention? | Y | Comment: One participant was removed from analysis due to missing one stimulation session. All other participants were analysed according to their group assignment. |  |
|  | 2.7 Was there potential for a substantial impact of the failure to analyse participants in the group to which they were randomised? | NA | - |  |
| 3. Missing outcome data | 3.1 Were data for this outcome available for all, or nearly all, participants randomised? | Y | Comment: Data is available for nearly all participants (n=1 not included in analyses). | Low |
|  | 3.2 Is there evidence that the result was not biased by missing outcome data? | NA | - |  |
|  | 3.3 Could missingness in the outcome depend on its true value? | NA | - |  |
|  | 3.4 Is it likely that missingness in the outcome depended on its true value? | NA | - |  |
| 4. Measurement of the outcome | 4.1 Was the method of measuring the outcome inappropriate? | N | Quote: “The HRV was recorded by a HR monitor (Polar^®^ RS800CX...”. See “2.5 LF, HF, and LF/HF ratio”, pg. 161.  Quote: “The LF/HF ratio, HF, and LF were calculated by the Kubios HRV software…”. See “2.5 LF, HF, and LF/HF ratio”, pg. 162. | Low |
|  | 4.2 Could measurement or ascertainment of the outcome have differed between interventions within each sequence? | N | Comment: Measurements were conducted the same for both groups. |  |
|  | 4.3 Were outcomes assessors aware of the intervention received by study participants? | PN | Comment: As per 2.1. |  |
|  | 4.4 Could assessment of the outcome have been influenced by knowledge of intervention received? | NA | - |  |
|  | 4.5 Is it likely that assessment of the outcome was influenced by knowledge of intervention received? | NA | - |  |
| 5. Selection of the reported result | 5.1 Analysed in accordance with a pre-specified analysis plan | NI | Comment: No pre-specified analysis plan. | High |
|  | Is the numerical result being assessed likely to have been selected, on the basis of the results, from… | - | - |  |
|  | 5.2 …multiple eligible outcome measurements (e.g. scales, definitions, time points) within the outcome domain? | NI |  |  |
|  | 5.3 …multiple eligible analyses of the data? | Y | Comment: See table 2, pg 163. Data logarithmically transformed for two-way ANOVA but not for one-way ANOVA. |  |
|  | 5.4 …carryover having been identified? | NI |  |  |
| Overall risk of bias | **High** | | | |

| ***Risk of bias*** | *Karri (2018)* |  |  |  |
| --- | --- | --- | --- | --- |
| **Domain** | **Signalling** | **Judgement** | **Support for judgement** | **Domain risk of bias** |
| 1. Randomisation process | 1.1 Random allocation sequence | NI | Quote: “All SCI+NP subjects participated in two experiments, which were administered in a random order, on different days, and at least 3 days apart.” See “Breathing only (null) and BreEStim (active) interventions”, pg. 2333.  Comment: Insufficient detail regarding random allocation sequence. | Some concerns |
|  | 1.2 Allocation concealment | NI | Comment: Insufficient detail regarding allocation concealment. |  |
|  | 1.3 Baseline differences | N | Comment: Baseline differences do not suggest an issue in the randomisation process. |  |
| S. Period and carryover effects | S.1 Was the number of participants allocated to each of the two sequences equal or nearly equal? | NI | Comment: Number of participants allocated to each of the two sequences is not reported. | Low |
|  | S.2 Were period effects accounted for in the analysis? | Y | Quote: “Two-way ANOVA tests and the Kruskal–Wallis tests with Scheirer–Ray–Hare extension were conducted to measure the interaction of time (BS and post-test) and treatment (null and active) factors in producing changes in parametric and non-parametric variables, respectively.” See “Statistical analyses”, pg. 2334. |  |
|  | S.3 Was there sufficient time for any carryover effects to have disappeared before outcome assessment in the second period? | Y | Comment: 72-hours appears to be sufficient time to avoid carry-over effects between stimulation sessions. |  |
| 2. Deviations from intended interventions | 2.1 Participants aware of assigned intervention | PY | Quote: “For Experiment 2 (active), subjects followed the same protocol for deep, voluntary breathing as described earlier. Additionally, a pair of trimmed surface electrodes ~2 cm×2 cm separated by ~1 cm was placed over the ventral aspect of the distal forearm along the path of the median nerve.” See “Breathing only (null) and BreEStim (active) interventions”, pg. 2333.  Comment: There is no mention of blinding in the paper. The nature of the intervention would make blinding difficult. | Some concerns |
|  | 2.2 Carers/people delivering interventions aware of assignment | PY | Comment: There is no mention of blinding in the paper. Given the nature of the intervention, blinding of carers/people delivering the interventions would be difficult |  |
|  | 2.3 Deviations from intended intervention due to trial context | NI | Comment: No mention of deviations from intended intervention due to trial context. |  |
|  | 2.4 Were these deviations likely to have affected the outcome? | NA | - |  |
|  | 2.5 Were these deviations from intended intervention balanced between groups? | NA | - |  |
|  | 2.6 Was an appropriate analysis used to estimate the effect of assignment to intervention? | Y | Comment: All participants were analysed in a manner appropriate for evaluating the effect of assignment. |  |
|  | 2.7 Was there potential for a substantial impact of the failure to analyse participants in the group to which they were randomised? | NA | - |  |
| 3. Missing outcome data | 3.1 Were data for this outcome available for all, or nearly all, participants randomised? | Y | Comment: Data for this outcome is available for all participants randomised. | Low |
|  | 3.2 Is there evidence that the result was not biased by missing outcome data? | NA | - |  |
|  | 3.3 Could missingness in the outcome depend on its true value? | NA | - |  |
|  | 3.4 Is it likely that missingness in the outcome depended on its true value? | NA | - |  |
| 4. Measurement of the outcome | 4.1 Was the method of measuring the outcome inappropriate? | N | Quote: “ECG recording was collected using a heart rhythm scanner  (Biocom 5000 Wireless ECG Recorder; Biocom Technologies,  Poulsbo, WA, USA). ECG heart rhythms were saved  for off-line HRV analysis.” See “VAS scoring and HRV collection”, pg. 2333.  “Kubios HRV analysis software (University of Eastern Finland, Joensuu, Finland) was used to evaluate the ECG recording.” See “Data analysis”, pg. 2334. | Low |
|  | 4.2 Could measurement or ascertainment of the outcome have differed between interventions within each sequence? | N | Comment: Measurements were conducted the same for both groups. |  |
|  | 4.3 Were outcomes assessors aware of the intervention received by study participants? | NI | Quote: “Sheng Li was blinded to all experiments and did not have direct patient contact in this study.” See “Disclosure”, pg. 2340.  Comment: No information provided as to whether SL was a blinded assessor. |  |
|  | 4.4 Could assessment of the outcome have been influenced by knowledge of intervention received? | PN | Comment: HRV is an observer-reported outcome. It is unlikely that is influenced due to knowledge of intervention received. |  |
|  | 4.5 Is it likely that assessment of the outcome was influenced by knowledge of intervention received? | NA | - |  |
| 5. Selection of the reported result | 5.1 Analysed in accordance with a pre-specified analysis plan | NI | Comment: No pre-specified analysis plan. | Some concerns |
|  | Is the numerical result being assessed likely to have been selected, on the basis of the results, from… |  |  |  |
|  | 5.2 …multiple eligible outcome measurements (e.g. scales, definitions, time points) within the outcome domain? | NI |  |  |
|  | 5.3 …multiple eligible analyses of the data? | NI |  |  |
|  | 5.4. …carryover having been identified? | NI |  |  |
| Overall risk of bias | **Some concerns** | | | |

| ***Risk of bias*** | *Ochiai (2017)* |  |  |  |
| --- | --- | --- | --- | --- |
| **Domain** | **Signalling** | **Judgement** | **Support for judgement** | **Domain risk of bias** |
| 1. Randomisation process | 1.1 Random allocation sequence | NI | Quote: “The order of the conditions… was randomized.” See “2.1 Experimental Design”, pg. 3  Comment: Insufficient detail regarding random allocation sequence. | Some concerns |
|  | 1.2 Allocation concealment | NI | Comment: Insufficient detail regarding allocation concealment. |  |
|  | 1.3 Baseline differences | N | Comment: Baseline differences do not suggest an issue in the randomisation process. |  |
| S. Period and carryover effects | S.1 Was the number of participants allocated to each of the two sequences equal or nearly equal? | NI | Comment: The study does not report on how many participants started in each group. | High |
|  | S.2 Were period effects accounted for in the analysis? | N | Comment: Period effects were not accounted for in the analysis. |  |
|  | S.3 Was there sufficient time for any carryover effects to have disappeared before outcome assessment in the second period? | N | Comment: No mention of wash-out period. Assumed that there was no wash-out period. |  |
| 2. Deviations from intended interventions | 2.1 Participants aware of assigned intervention | PY | Comment: No mention of blinding. It would be difficult to blind participants with this type of intervention. | Some concerns |
|  | 2.2 Carers/people delivering interventions aware of assignment | PY | Comment: No mention of blinding. |  |
|  | 2.3 Deviations from intended intervention due to trial context | NI | Comment: No mention of deviations from intended intervention due to trial context. |  |
|  | 2.4 Were these deviations likely to have affected the outcome? | NA | - |  |
|  | 2.5 Were these deviations from intended intervention balanced between groups? | NA | - |  |
|  | 2.6 Was an appropriate analysis used to estimate the effect of assignment to intervention? | NI | Comment: The number of participants analysed was not explicitly stated. |  |
|  | 2.7 Was there potential for a substantial impact of the failure to analyse participants in the group to which they were randomised? | PN | - |  |
| 3. Missing outcome data | 3.1 Were data for this outcome available for all, or nearly all, participants randomised? | NI | Comment: See 2.6. | Low |
|  | 3.2 Is there evidence that the result was not biased by missing outcome data? | N | - |  |
|  | 3.3 Could missingness in the outcome depend on its true value? | PN | - |  |
|  | 3.4 Is it likely that missingness in the outcome depended on its true value? | NA | - |  |
| 4. Measurement of the outcome | 4.1 Was the method of measuring the outcome inappropriate? | N | Quote: “The patients placed their left forefingers on the sensor of an accelerated plethysmograph…. Heart rate variability (HRV) was analyzed.” See “2.2 Physiological Indices”, pg. 3 | Low |
|  | 4.2 Could measurement or ascertainment of the outcome have differed between interventions within each sequence? | N |  |  |
|  | 4.3 Were outcomes assessors aware of the intervention received by study participants? | NI | Comment: No mention of blinding. |  |
|  | 4.4 Could assessment of the outcome have been influenced by knowledge of intervention received? | PN | Comment: HRV is an observer-reported outcome. It is unlikely that is influenced due to knowledge of intervention received. |  |
|  | 4.5 Is it likely that assessment of the outcome was influenced by knowledge of intervention received? | NA | - |  |
| 5. Selection of the reported result | 5.1 Analysed in accordance with a pre-specified analysis plan | NI | Comment: No pre-specified analysis plan | Some concerns |
|  | Is the numerical result being assessed likely to have been selected, on the basis of the results, from… |  |  |  |
|  | 5.2 …multiple eligible outcome measurements (e.g. scales, definitions, time points) within the outcome domain? | NI |  |  |
|  | 5.3 …multiple eligible analyses of the data? | NI |  |  |
|  | 5.4. …carryover having been identified? | NI |  |  |
| Overall risk of bias | **High** | | | |
