# Supplementary material for: The Effect of Non-Invasive, Non-Pharmacological Interventions on Autonomic Regulation of Cardiovascular Function in Adults with Spinal Cord Injury: A Systematic Review with Meta-Analysis
Source: Neurotrauma Rep. 2025 Jan 13;5(1):1151–72. doi: 10.1089/neur.2024.0110 (PMC11848056; doi:10.1089/neur.2024.0110)
Supplement: Supplementary Table S8 [file neur.2024.0110_supp_table8.docx]

| **Table S8:** Details of amendments made to the prospectively registered protocol. | | |
| --- | --- | --- |
| **Statement made in protocol** | **Amendment to the protocol** | **Rationale for amendment** |
| “Grey literature, unpublished studies and dissertations will also be included.” | Grey literature, unpublished studies, and dissertations were not included. | These literature forms can often have quality concerns and be difficult to access. To ensure the review included high quality literature, these literature forms were excluded. |
| “We may also include studies that are cohort longitudinal with an intervention” | Only RCTs, crossover trials, and controlled trials were included. | Without a randomised component, studies introduce a substantial risk of bias. To improve the quality of the evidence included in the review, only studies with a randomised component were included. |
| “Studies without a control or able-bodied group will be excluded in statistical analysis but reported separately elsewhere.” | Participants with SCI were only included as a control group. These were not reported separately. | An able-bodied group does not provide a fair comparison to adults with SCI when evaluating the effect of interventions on autonomic function in an RCT. |
| “Main outcome(s): Heart Rate Variability” | SBPV-LF and BR gain were included as primary outcomes. Only RMSSD, HRV-LF, HRV-HF, SBPV-LF, and BR gain were included as primary outcomes. | Whilst HRV is a valuable indicator of cardiac vagal activity, it is not as effective in quantifying cardiac sympathetic activity. SBPV-LF was included as it is a good indicator of cardiovascular sympathetic activity. BR gain provides an indication of the interact The physiological mechanisms behind other HRV metrics (i.e., total power, non-linear measures) are not well established and were removed as primary outcomes. LF/HF and non-linear outcomes were removed as outcomes given interpretations of these metric are not as clear. |
| “Only Heart Rate Variability recordings of >5 minutes will be included” | Studies with recordings <5 minutes were included. | Whilst HRV guidelines recommend at least 5-minute recordings, studies have shown HRV to be valid for as little as 20 second recordings. |
| “Additional outcomes(s)… Heart rate, blood pressure, …. respiration rate.” | These were not included as secondary outcomes. Quality of life and adverse events were added as secondary outcomes. | Heart rate, blood pressure, and respiration rate were deemed not required for this review. Quality of life was included as it is an indicator of overall well-being of an individual in relation to their context. This is valuable as it reflects the direct impact of a SCI on an individual and how interventions are able to minimise this impact. Additionally, capturing adverse events helps to evaluate the safety of an intervention. |
| BPV: Blood pressure variability; BR: baroreflex; HRV: Heart rate variability; RCT: Randomised controlled trial; SBPV: Systolic blood pressure variability; SCI: Spinal cord injury. | | |
